# αSynuclein control of mitochondrial homeostasis in human-derived neurons is disrupted by mutations associated with Parkinson’s disease

**DOI:** 10.1038/s41598-017-05334-9

**Published:** 2017-07-11

**Authors:** Victorio Martin Pozo Devoto, Nicolas Dimopoulos, Matías Alloatti, María Belén Pardi, Trinidad M. Saez, María Gabriela Otero, Lucas Eneas Cromberg, Antonia Marín-Burgin, Maria Elida Scassa, Gorazd B. Stokin, Alejandro F. Schinder, Gustavo Sevlever, Tomás Luis Falzone

**Affiliations:** 1 Instituto de Biología Celular y Neurociencias, IBCN (UBA-CONICET), Facultad de Medicina, Universidad de Buenos Aires, Paraguay 2155, Buenos Aires, CP1121 Argentina; 2 International Clinical Research Center (ICRC), St. Anne’s University Hospital, CZ-65691 Brno, Czech Republic; 3 Fundación para la Lucha contra las Enfermedades Neurológicas de la Infancia (FLENI), Montañeses 2325, Buenos Aires, C1428AQK Argentina; 4 Instituto de Biología y Medicina Experimental, IBYME (CONICET). Vuelta de obligado 2490, Buenos Aires, CP 1428 Argentina; 5 Instituto de Investigación en Biomedicina de Buenos Aires (IBioBA) –CONICET - Partner Institute of the Max Planck Society, Buenos Aires, Argentina; 6 Laboratorio de Plasticidad Neuronal, Fundación Instituto Leloir (IIBBA – CONICET), Av. Patricias Argentinas 435, Buenos Aires, CP C1405BWE Argentina

## Abstract

The etiology of Parkinson’s disease (PD) converges on a common pathogenic pathway of mitochondrial defects in which α-Synuclein (αSyn) is thought to play a role. However, the mechanisms by which αSyn and its disease-associated allelic variants cause mitochondrial dysfunction remain unknown. Here, we analyzed mitochondrial axonal transport and morphology in human-derived neurons overexpressing wild-type (WT) αSyn or the mutated variants A30P or A53T, which are known to have differential lipid affinities. A53T αSyn was enriched in mitochondrial fractions, inducing significant mitochondrial transport defects and fragmentation, while milder defects were elicited by WT and A30P. We found that αSyn-mediated mitochondrial fragmentation was linked to expression levels in WT and A53T variants. Targeted delivery of WT and A53T αSyn to the outer mitochondrial membrane further increased fragmentation, whereas A30P did not. Genomic editing to disrupt the N-terminal domain of αSyn, which is important for membrane association, resulted in mitochondrial elongation without changes in fusion-fission protein levels, suggesting that αSyn plays a direct physiological role in mitochondrial size maintenance. Thus, we demonstrate that the association of αSyn with the mitochondria, which is modulated by protein mutation and dosage, influences mitochondrial transport and morphology, highlighting its relevance in a common pathway impaired in PD.

## Introduction

Parkinson’s disease (PD), the second most prevalent neurodegenerative disorder, is pathologically characterized by progressive neuronal loss and the accumulation of eosinophilic intracellular inclusions, termed Lewy bodies^1^. The major component of these inclusions is α-Synuclein (αSyn)^2^, the first protein linked with dominant familial PD^3^. So far, more than 20 loci are known to contribute to familial PD, many of which are implicated in the regulation of mitochondrial homeostasis^4, 5^. Although genetic mutations account for a small proportion of PD cases, there is both pathological and pharmacological evidence to support a key role for mitochondrial dysfunction in the progression of sporadic PD^6, 7^. Recent evidence supports a direct link between αSyn function and mitochondrial pathologies^8–10^; however, the precise molecular mechanisms by which αSyn may induce mitochondrial defects remain unclear.

Mitochondrial-related proteins govern both fusion-fission rates and transport, leading to a direct effect on mitochondrial size and homeostasis. Impairments in these processes have been associated with many neurodegenerative diseases^11^. In PD, familial mutations in PTEN-induced putative kinase 1 (PINK1), Parkin, DJ-1, leucine-rich repeat kinase (LRRK), ATP13A2, and recently, vacuolar protein sorting-associated protein 35 (VPS35) have been found to control mitochondrial size and transport through interaction with fusion-fission proteins and molecular motors^12–18^.

Patients carrying αSyn gene (*SNCA*) triplications display earlier disease onset compared with patients carrying *SNCA* duplications, indicating that intracellular levels of αSyn determine the development of pathology in a dose-dependent manner^19, 20^. In addition to the aggregation properties of αSyn, *in vitro* membrane-binding studies suggest that αSyn adopts an α-helix conformation at the N-terminal domain when interacting with lipids^21^. Disruption of this conformation via the A30P αSyn mutation leads to a reduced affinity for lipids, whereas the A53T αSyn mutation produces the opposite effect^22^. This difference in lipid affinity correlates with an earlier onset and more severe manifestations in patients with A53T mutation, while A30P induces late age onset and milder symptoms^23, 24^. However, it is unclear how these two distinct mutations, which have opposing effects on membrane affinity, result in mitochondrial defects.

Differential associations of αSyn mutants with mitochondria have been proposed, with some studies reporting the presence of αSyn inside the mitochondria, whereas others report localization to the outer mitochondrial membrane (OMM)^25–27^. Interestingly, changes in mitochondrial morphology were described as common features in several animal models of αSyn mutation or overexpression^28–30^ as well as in human cellular models^26, 31^. Moreover, mitochondrial fission has been linked to αSyn function^29, 31^. However, robust evidence demonstrating a direct regulation of mitochondrial size by αSyn-dependent mechanisms in human neurons is lacking.

Here, we sought to determine whether αSyn protein plays a direct role in the regulation of mitochondrial homeostasis, both under physiological and pathological conditions, in a human cell model. Using human neurons derived from embryonic stem cells (hESC) or induced pluripotent stem cells (hiPSC), we studied mitochondrial axonal transport and morphology in the presence of overexpressed wild-type or mutant αSyn. αSyn overexpression was found to induce mitochondrial transport defects and fragmentation. Forced delivery of αSyn to the OMM resulted in a strong reduction in mitochondrial size, suggesting that αSyn variants can induce differential effects in a common pathological pathway. Moreover, disruption of the α-helix conformation in the N-terminal domain of αSyn in human neurons uncovered a physiological function for endogenous αSyn in the maintenance of neuronal mitochondrial size. Our findings, in a relevant human neuronal model, demonstrate a key role for αSyn in the development of mitochondrial pathology associated with PD, and highlight a potential target for early therapeutic intervention prior to neuronal loss and clinical manifestation of PD.

## Results

### αSyn overexpression in hESC-derived neurons

**Figure 1:**
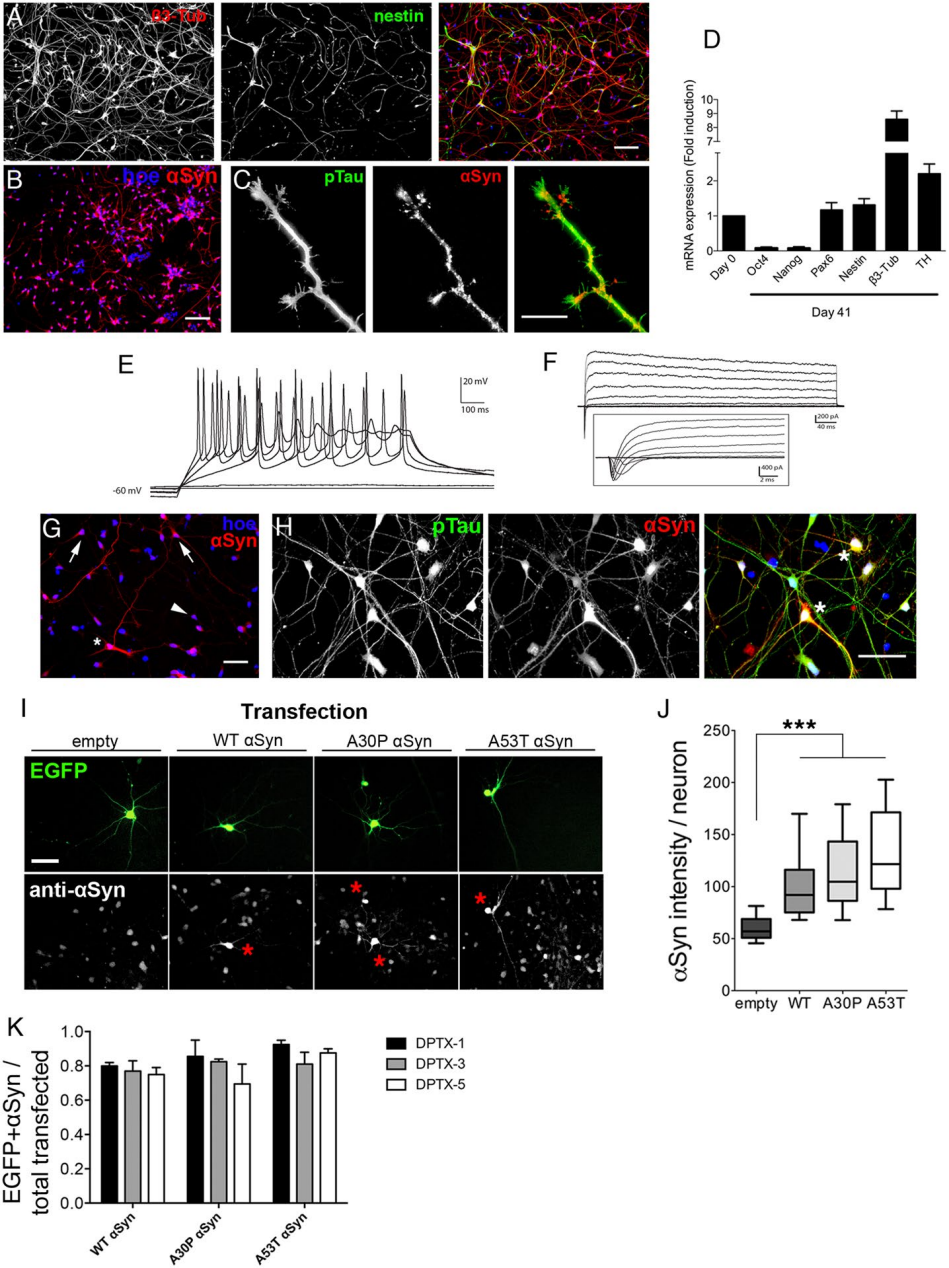
αSyn overexpression in functional hESC-derived neurons. (**A**–**F**) Human neuronal culture characterization. (**A**) High expression of the neuronal marker, β3-tubulin, and low expression of the precursor marker, nestin (Scale bar, 100 μm). (**B**) Endogenous αSyn cytosolic localization. Nuclei stained with Hoechst (blue). Scale bar, 100 μm. (**C**) αSyn localization is enriched within neuronal processes (p-Tau). Scale bar, 20 μm. (**D**) qPCR quantification at day 41 of the differentiation protocol showing mRNA expression for stemness (Oct4, Nanog), neural markers (Pax6, Nestin), and specific neuronal markers (Tuj1, TH) relative to undifferentiated hESC (mean ± SEM, n = 4 independent experiments). (**E**) Representative traces of spiking evoked by stepwise increases of current from 0 to 40 pA, incremented by 10 pA. Cells were kept at −60 mV in current clamp. (**F**) Representative traces of Na+ and K+ voltage-dependent currents evoked by depolarizing voltage steps, incremented every 10 mV. Cells were voltage clamped at −70 mV. Inset shows higher temporal resolution. (**G**–**J**). Human neurons overexpressing αSyn. (**G**) αSyn immunofluorescent staining in transfected neurons (asterisk), non-transfected neurons (arrows), and non-αSyn-expressing cells (arrowhead) at DIV14 cultures. Scale bar, 50 μm. (**H**) Neurons (p-tau) transfected with αSyn display increased levels of expression by immunofluorescence. Scale bar, 50 μm. (**I** and **J**) αSyn immunofluorescent staining in neurons co-transfected with EGFP and control (empty), WT, A30P, or A53T αSyn (asterisks). Scale bar, 50 μm. Intensity measurements of αSyn staining in the different conditions. Data represented as median plus interquartile range. Kruskal-Wallis ANOVA (p < 0.05). (n = 80 neurons per condition from 2 independent experiments). (**K**) Average cell ratio performed to identify neuron survival upon αSyn expression in 1, 3 and 5 days post-transfection (DPTX). Ratio obtained by dividing αSyn positive neurons (overexpressing above threshold in Fig. S3C) by total EGFP + neurons (above and below αSyn threshold) (n = 20 images per condition, more than 5 neurons by field from 2 independent experiments). See also Figures S1, S2 and S3.

To study the physiological and pathological roles of αSyn in mitochondrial dynamics within the context of human PD, we obtained enriched, polarized, and functional human neurons derived from hESC according to a previously described protocol (Figs 1A–F, S1A)^32^. The differentiation stages from pluripotent colonies to terminally differentiated neurons were evaluated by quantification of mRNA and protein expression levels. Pluripotent markers (OCT4, SOX2, Nanog) were present in the undifferentiated stages (Figure S1B) and downregulated at final differentiation, whereas neuronal markers, including βIII tubulin, pan-NCAM, and tyrosine hydroxylase (TH), appeared upregulated in differentiated neurons (Figs 1D, S1B and S1C). Imaging of differentiated neurons indicated conspicuous expression of βIII tubulin but weak expression of nestin (Fig. 1A). A diffuse cytosolic pattern of endogenous αSyn was observed, with punctate distribution among neurites labeled with p-Tau (Fig. 1B and C). Furthermore, enriched neuronal cultures (55.17%+/− 2.2; Figure S3A) showed a time-dependent maturation of tau isoform splicing^33^ and progression of electrophysiological profiles, including repetitive spiking and increased amplitude of voltage-dependent Na^+^ and K^+^ currents (Figs 1E,F and S2), recapitulating key hallmarks in neuronal development. To examine the role of αSyn in mitochondria, neurons were transfected with pLV-CMV-αSyn vectors encoding wild-type (WT) αSyn or the familial PD mutations, A30P or A53T (Fig. 1G). Based on electrophysiological profile and robust neuronal marker expression indicative of neuronal differentiation, transfections were performed on day 14 *in vitro* (DIV14). Increased expression of αSyn was observed in transfected p-Tau-labeled neurons (Fig. 1H). To distinguish transfected αSyn from endogenous protein, neurons co-transfected with vectors encoding EGFP and WT, A30P, or A53T αSyn were analyzed by immunofluorescence (Fig. 1I). Quantification of αSyn fluorescent intensity revealed a 2- to 3-fold increase in αSyn signal in transfected neurons (WT, A30P, or A53T) compared with endogenous αSyn expression (Fig. 1J), characterizing αSyn overexpression profiles in our neuronal model. αSyn overexpression was further validated by flow cytometric analysis of neurons transfected with vectors encoding mCherry and WT, A30P, or A53T αSyn (Figure S3B). No neurotoxic effects were observed in response to overexpression of any of the αSyn variants (Figs 1K, S3C).

### αSyn overexpression induces mitochondrial axonal transport defects

**Figure 2:**
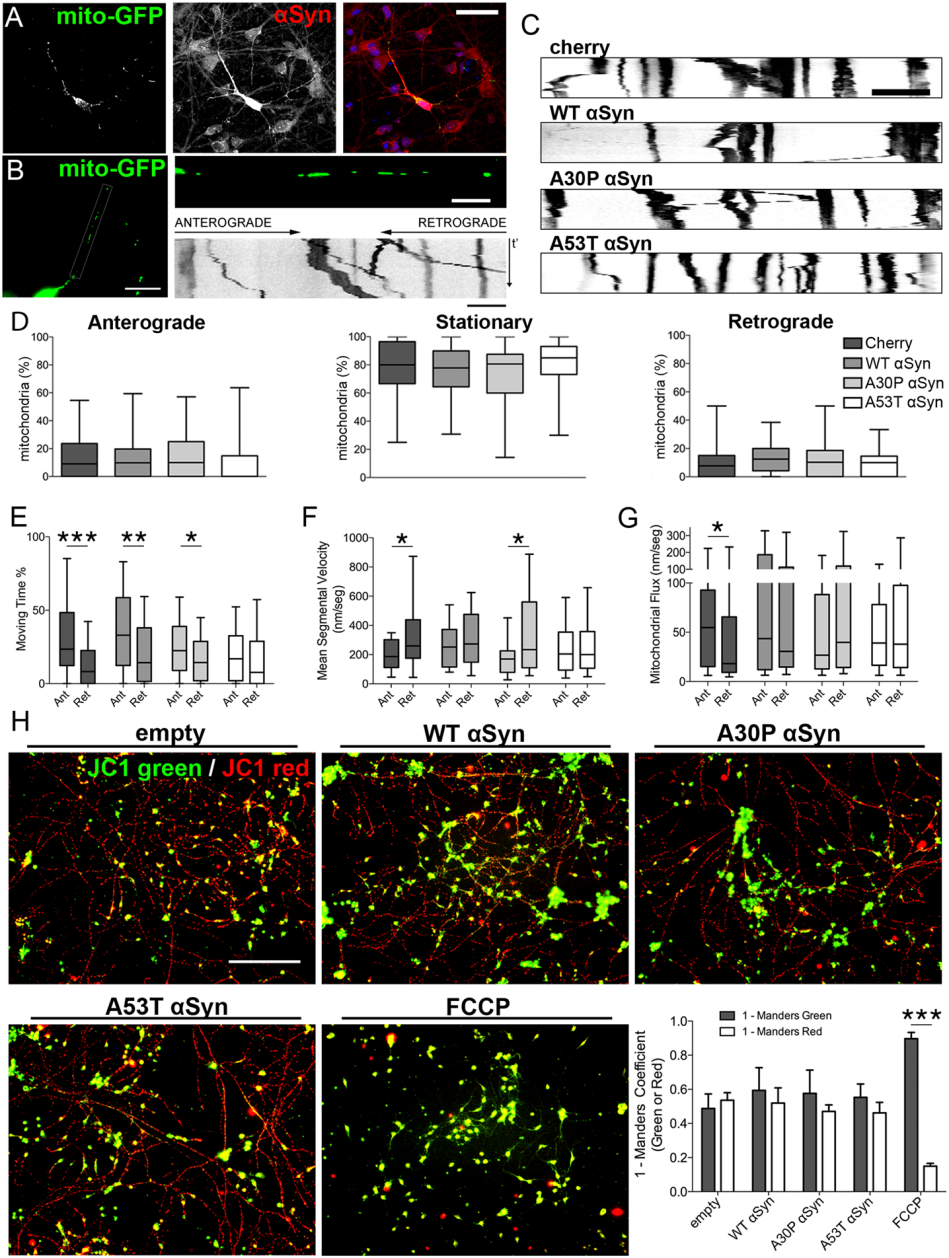
Mitochondrial axonal transport is differentially affected by αSyn variants. (**A**) Neuron co-transfected with mito-GFP and WT αSyn. Scale bar, 100 μm. (**B**) Mitochondria (mito-GFP) in axonal projections from a transfected neuron used for live-imaging, magnified projection, and kymograph plotting. Kymograph obtained from a 150-s time-lapse movie at 3-s intervals. Left panel scale bar: 30 μm, horizontal: 10 μm. (**C**) Representative kymographs for αSyn transfected neurons obtained from supplementary movies (Sup Movies 1–4). Scale bar, 10 μm. (**D**) Overall anterograde, retrograde, or stationary axonal mitochondrial proportion does not change upon αSyn overexpression. Data represented as median plus interquartile range (n > 50 axons per condition from 10 independent experiments). Kruskal-Wallis ANOVA for each type of movement (p > 0.05). (**E**) Anterograde to retrograde moving time differences observed in control mitochondria are lost in A53T transfected neurons but not in WT and A30P αSyn. (**F**) Anterograde to retrograde mean segmental velocity differences are lost in WT and A53T αSyn-transfected neurons (n = 60 per condition from 10 independent experiments). (**G**) Anterograde to retrograde mitochondrial flux (traveled distance per mitochondria normalized to acquisition time (nm/seg)) is impaired in all αSyn overexpression conditions compared with control (n = 60 per condition from 10 independent experiments). Data represented as median plus interquartile range. Mann-Whitney U test between anterograde and retrograde movements for each parameter. ***p < 0.001, **p < 0.01, *p < 0.05. (**H**) αSyn overexpression does not impair mitochondrial membrane potential. Mitochondrial membrane potential was analyzed in transduced DIV14 neurons using JC1. Low and high (green and red emission, respectively) membrane potential was quantified by fluorescence intensity and Manders’ colocalization coefficient was calculated. Mitochondrial ionophore FCCP was used as positive control. Data represented as mean ± SEM (n = 7 independent experiments). Two-way ANOVA followed by Sidak’s corrected comparison of Manders’ coefficient for each condition, ***p > 0.001. Scale bar, 200 μm. See also Movies M1–M4, Figure S4.

Neuronal homeostasis is reliant on the appropriate subcellular distribution of organelles, including mitochondria^18, 34^. αSyn expression has been shown to influence axonal transport, which in turn affects mitochondrial fusion-fission rates^13, 17, 35, 36^. To test the effects of αSyn overexpression on axonal transport of mitochondria in our human-derived neuronal model, we performed live-imaging analysis of cells co-transfected with a vector driving GFP fused to a mitochondrial-targeting sequence (mito-GFP) in addition to WT, A30P, or A53T αSyn-expressing vectors (Figs 2A, S4A). Time-lapse movies from transfected axons were transformed to kymographs to track and quantify mitochondrial movement (Fig. 2B, Movies M1–4). Mitochondrial transport from WT, A30P, or A53T αSyn (Fig. 2C) showed similar overall anterograde, stationary, and retrograde mitochondrial proportions when compared with control (mCherry-transfected) neurons (Fig. 2D). However, detailed analysis of mitochondrial kinetics, by discriminating segmental velocities and time during movement (Fig. 2E and F), revealed a difference in control neurons, which displayed a significant bias to anterograde flux compared with retrograde flux (Fig. 2G). This asymmetry, which is important for maintenance of distal distribution of axonal mitochondria, was lost in αSyn-overexpressing cells (Fig. 2G). WT and A53T αSyn-overexpressing neurons showed similar anterograde and retrograde mitochondrial segmental velocities (Fig. 2F), and A53T αSyn overexpression also resulted in similar time spent moving in retrograde and anterograde directions (Fig. 2E). These results reveal impairments in mitochondrial flux in transfected axons, with the most pronounced effect in A53T αSyn-overexpressing neurons, indicating that αSyn overexpression leads to an imbalance in the distribution of mitochondria throughout the axon.

To test whether mitochondrial flux impairments are related to an αSyn effect on mitochondrial homeostasis, we assessed mitochondrial membrane potential in neurons transduced with WT, A30P, or A53T pLV-CMV-αSyn (Figs 2H, S4B). JC1, a mitochondrial probe that changes its emission wavelength when oligomerized under hyperpolarizing membrane potential, was used to assess mitochondrial function. Colocalization analysis using Manders’ correlation coefficient for red versus green intensities showed no significant changes in mitochondrial membrane potential in any of the αSyn-overexpressing neurons (Figs 2H and S4C). Our results indicate that axonal flux impairments observed upon αSyn overexpression are not caused by depolarization of the mitochondria; thus, the effects seen are unlikely to be the result of short-term effects on mitochondrial health.

### Axonal mitochondrial fragmentation in neurons overexpressing A53T αSyn

**Figure 3:**
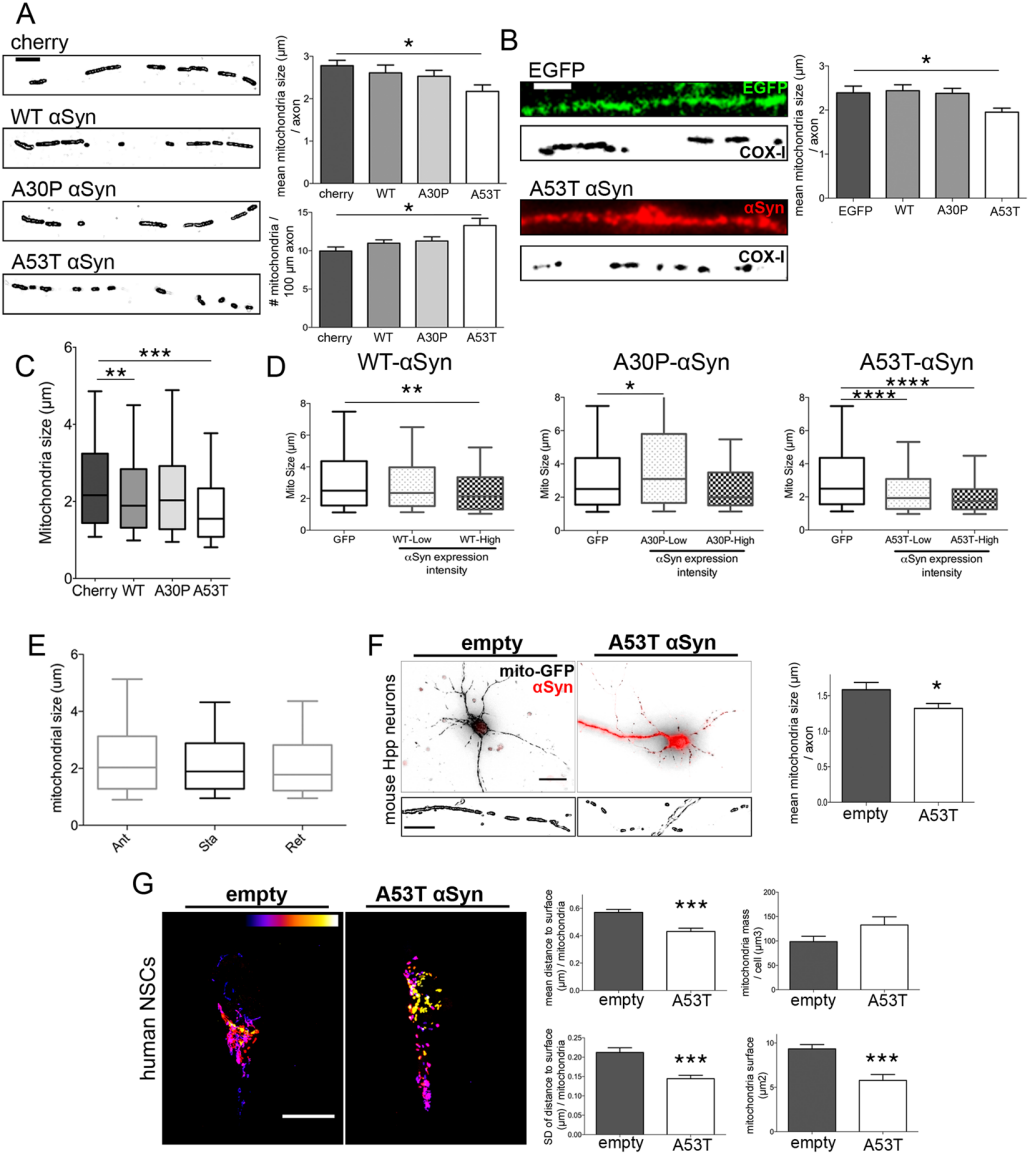
A53T αSyn overexpression reduces mitochondrial size and increases density in human neurons. (**A**) Mean size and density of mitochondria in axons from WT, A30P, or A53T αSyn and mito-GFP co-transfected neurons (n = 60 per condition from 10 independent experiments). Scale bar, 5 μm. (**B**) A53T αSyn-induced mitochondrial size reductions were confirmed using immunofluorescent staining against COX-I in transfected neurons. Data is represented as mean ± SEM. One-way ANOVA followed by Dunnett’s post-test, *p < 0.05 (n = 40 from 4 independent experiments). Scale bar, 5 μm. (**C**) A53T and, to a lesser extent, WT αSyn reduced the size of mitochondria. Data presented as median plus interquartile range. (**D**) Median and percentiles corresponding to mitochondrial size categorized by low or high αSyn (above αSyn maximum intensity of GFP only-transfected cells) fluorescence intensity on each treatment. Kruskal-Wallis ANOVA for low and high αSyn variant compared with control (p < 0.05) (n = 20 neurons). (**E**) Average mitochondrial size from different conditions pooled together for anterograde, stationary, and retrograde mitochondria. Data represented as median, 25 and 75 percentiles box, 10 and 90 percentiles whiskers. No significant differences were found. Kruskal-Wallis ANOVA (p > 0.05). (n = 2000 mitochondria from 10 independent experiments). (**F-G**) A53T αSyn effects on mitochondrial size are not limited to human neurons. (**F**) Mouse hippocampal neurons co-transfected with mito-GFP and empty vector or A53T αSyn. Inset: magnification of the axon. Scale bar, 30 μm, inset scale bar, 10 μm. Mean mitochondrial size quantification in axons from A53T αSyn-overexpressing mouse neurons compared with control (n = 30 axons from 3 independent experiments). (**G**) Three-dimensional analysis of mitochondrial morphology in human neural stem cells co-transfected with mito-GFP and empty vector or A53T αSyn. Color-coded bar shows mitochondrial location in z-axis. Bar graphs show reduction in mitochondrial surface, mean distance to surface, and standard deviation (SD) of distance to surface with no changes in total mass upon A53T αSyn overexpression (n = 15 cells from 2 independent experiments). Scale bar, 20 μm. Data is represented as mean ± SEM. Paired t-test, ***p < 0.001, *p < 0.05. See also Figure S5.

To test whether the changes in mitochondrial flux observed upon αSyn overexpression in human neurons promote mitochondrial fragmentation, as previously described in both cellular and animal systems^28–31^, we analyzed the morphology of axonal mitochondria after co-transfecting human-derived neurons with mito-GFP and αSyn vectors (Fig. 3A). Control, WT, and A30P αSyn-overexpressing neurons showed similar mean mitochondrial lengths per axon. However, transfection with A53T αSyn resulted in a significant decrease in mitochondrial length and an increased number of mitochondria per axon (Fig. 3A). To confirm this result, an immunofluorescent staining approach using mitochondrial cytochrome C oxidase I (COX-I) was performed in neurons transfected with EGFP, WT, A30P, or A53T αSyn vectors. Again, A53T αSyn overexpression resulted in a significant reduction in the size of axonal mitochondria while no changes were observed upon WT or A30P αSyn overexpression (Fig. 3B). In addition, total axonal mitochondria exhibited a significant size decrease in A53T and WT αSyn-transfected neurons (Fig. 3C). To test whether levels of αSyn expression correlated with fragmentation phenotype, co-transfected neurons were evaluated for mitochondrial size and αSyn intensity (Fig. 3D). Interestingly, low αSyn-expressing neurons showed significant fragmentation only in A53T αSyn (Fig. 3D), whereas high αSyn-expressing neurons showed significant mitochondrial fragmentation phenotypes in A53T and WT αSyn, but no difference in A30P compared with control (GFP, Fig. 3D). These results suggest that different αSyn variants induce a dose-dependent mitochondrial fragmentation effect. We then considered whether mitochondrial size correlated with movement, given the observed changes in mitochondrial flux and the possibility that smaller mitochondria may be more prone to movement. However, no association between mitochondrial size and type of mitochondrial movement was observed (Fig. 3E).

To determine whether the effect of A53T αSyn on mitochondrial size was specific to human-derived neurons, A53T αSyn was overexpressed in mouse primary neuronal cultures and in human neural stem cells (hNSCs). Consistent with our earlier results, co-transfection of mito-GFP and A53T αSyn into mouse hippocampal neurons resulted in a significant reduction in axonal mitochondrial length when compared with control (Fig. 3F). A significant decrease in mitochondrial size was also evident in hESC-derived NSCs upon A53T αSyn overexpression (Figs 3G and S5). Three-dimensional structural reconstruction analysis of mitochondria from transfected hNSCs showed that A53T αSyn not only led to a significant size reduction (surface and mean distance to surface), but also resulted in alterations in morphology, with mitochondrial shape becoming highly spherical (standard deviation of distance, Fig. 3G). Moreover, total mitochondrial mass was unaltered, indicating a compensatory increase in mitochondrial number (Fig. 3G). Taken together, these results reveal that A53T αSyn impairs normal mitochondrial homeostasis, inducing mitochondrial fragmentation in both neuronal and non-neuronal cells.

### Targeted mitochondrial delivery of WT and A53T αSyn, but not A30P, reduces mitochondrial size

**Figure 4:**
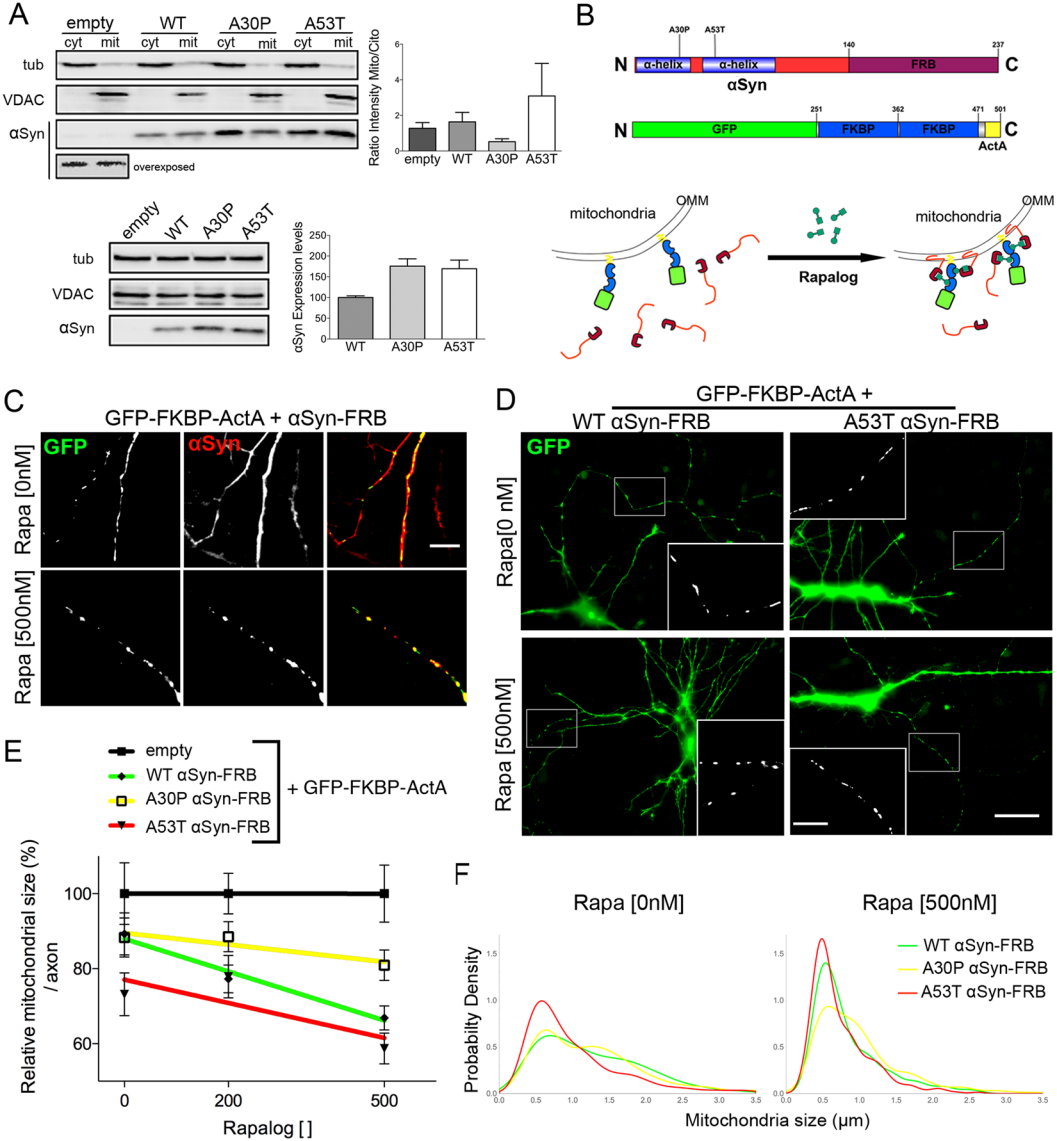
Differential αSyn association to mitochondrial membrane induces fragmentation. (**A**) Total, cytosolic, and mitochondrial fractions isolated by differential centrifugation of SHSY5Y cells transfected with empty, WT, A30P, or A53T αSyn vectors. Tubulin and VDAC1 were used as controls for cytosolic and mitochondrial fractions, respectively. Upper panel: αSyn enrichment quantified by mitochondrial/cytosolic ratio for control (empty), WT, A30P, or A53T αSyn normalized to the level of αSyn expression. See Figure S8 for complete overexposed gel. Lower panel: immunoblots showing total protein levels from homogenates and quantification of αSyn overexpression levels normalized to WT αSyn. Data is represented as mean ± SEM (n = 3 independent experiments). (**B**) Dimerization scheme showing fusion protein design to deliver αSyn to mitochondria. WT, A30P, or A53T αSyn were fused in-frame to FRB motif (αSyn-FRB). A vector driving two FKBP domains fused to GFP and an OMM peptide (GFP-FKBP-ActA) that localize to mitochondria was used. FRB and FKBP dimerize in the presence of rapalog, inducing αSyn-FRB delivery to the OMM. (**C**–**F**) WT and A53T, but not A30P αSyn, targeting to the OMM induces mitochondrial fragmentation. (**C**) Human neurons co-transfected with GFP-FKBP-ActA and αSyn-FRB were immunostained for GFP and αSyn to observe the αSyn cytosolic localization in 0 nM rapalog and the mitochondrial delivery in the presence of 500 nM rapalog for 6 h (Scale bar, 10 μm). (**D**) Neurons co-transfected with WT or A53T αSyn-FRB plus GFP-FKBP-ActA in the presence of 0 or 500 nM rapalog. Inset: axonal magnification. Scale bar, 30 μm; inset, 10 μm. (**E**) Mean mitochondrial size quantification per axon in 0, 200, or 500 nM rapalog for 6 h normalized to empty vector control. Linear regression, slopes, and deviation from zero were assessed (WT αSyn-FRB: −0.043, p < 0.001; A30P αSyn-FRB: −0.015, p > 0.05; A53T αSyn-FRB: −0.031, p < 0.05). Data is presented as mean ± SEM (n = 15 axons per condition from 3 independent experiments). (**F**) Probability function distribution of total pooled mitochondrial size measured in WT, A30P, or A53T αSyn-FRB plus GFP-FKBP-ActA neurons in 0 or 500 nM rapalog. (n > 300 mitochondria from 3 independent experiments). See also Figure S6.

The location of disease-associated αSyn mutations in the N-terminal domain of αSyn combined with the known biophysical interaction of this domain with lipid membranes led us to consider whether A53T αSyn might display enhanced membrane binding affinity^22, 37^. To determine whether αSyn variants differentially associate with the mitochondria, subcellular fractions (total, cytosolic, and mitochondrial) were obtained from a human neuroblastoma SH-SY5Y cell line transfected with WT, A30P, or A53T αSyn-expressing vectors (Fig. 4A). Quantification of αSyn subcellular distribution, calculated as the normalized mitochondrial/cytosolic ratio, showed a mild association of WT αSyn with the mitochondria, a reduction of A30P αSyn, and high enrichment in A53T αSyn when compared with control (Fig. 4A). Taken together, these results suggest that structural features of αSyn variants differentially determine their association with mitochondria and, consequently, the degree of mitochondrial defect.

To test whether the effect exerted by αSyn on mitochondrial size requires a direct association of αSyn with mitochondrial membranes, we designed a quantitative and time-dependent mitochondrial delivery experiment using the FKBP-FRB dimerization system^38^. WT, A30P, or A53T αSyn subcloned in frame to the FRB domain (WT, A30P, or A53T αSyn-FRB) dimerize with the FKBP domain in the presence of a heterodimerizing agent (rapalog). GFP and the actin assembly-inducing protein (ActA) fused to FKBP then target the dimerization complex to the OMM (Fig. 4B). GFP-FKBP-ActA mitochondrial localization and αSyn-FRB distribution with or without rapalog was assessed (Figs 4C, S6A and B). Two rapalog concentrations (200 and 500 nM) were tested to take into account dose-responsive effects. Consistent with our previous results, only A53T αSyn-FRB led to a significant reduction in mitochondrial size in the absence of rapalog (Figs 4D,E, and S6C). Rapalog incubation in A53T αSyn-FRB resulted in further reductions in the length of axonal mitochondria. Noteworthy, in the presence of 500 nM rapalog, WT αSyn-FRB led to a strong and significant reduction in the size of mitochondria similar to that seen with A53T αSyn-FRB (Figs 4D,E and S6C). In contrast, no effect on mitochondrial size in A30P αSyn-FRB neurons was observed even in the presence of the highest rapalog concentration (Fig. 4D). Consistent with this, probability density function analysis of mitochondrial size showed similarities between WT-FRB and A30P-FRB αSyn in the absence of rapalog, and between WT-FRB and A53T-FRB αSyn after 6-h incubation with 500 nM rapalog (Fig. 4F). Our results therefore strongly suggest a biophysical effect of αSyn on axonal mitochondrial fragmentation that requires a direct interaction with the OMM in neurons.

### αSyn N-terminal domain integrity is necessary for the control of neuronal mitochondrial morphology

**Figure 5:**
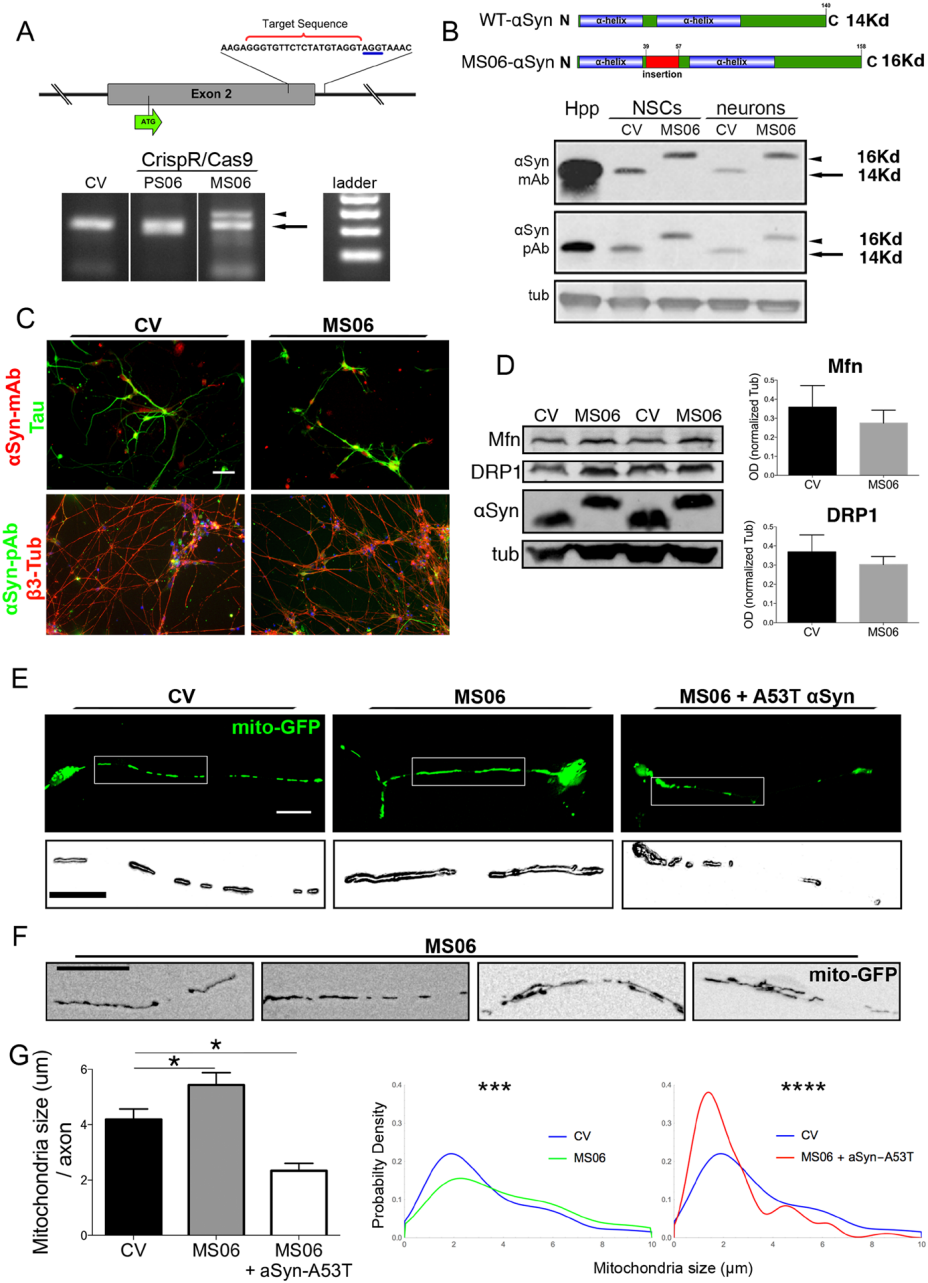
αSyn α-helix domain disruption increases mitochondrial size in human neurons. (**A**) Upper panel: Scheme of αSyn exon 2 sequence and guide RNA target sequence (red bracket) followed by the PAM sequence (blue) for CRISP-R/Cas9-mediated DNA cut. Lower Panel: Genomic PCR screening for insertions or deletions in CV hiPSC lines after CRISP-R/Cas9 edition. Two clones, PS06 (smaller size band) and MS06 (larger size band, arrowhead) are shown together with WT amplification (arrow). (**B**) Western blots from homogenates of mouse hippocampus (Hpp) and human neural stem cells or differentiated neurons from control or MS06 using two different antibodies against αSyn show a higher molecular weight αSyn in MS06 due to the insertion without downstream changes. Monoclonal (antigen aa 15–123) and polyclonal (antigen aa 111–131) anti-αSyn. (**C**) αSyn expression in terminally differentiated neurons derived from hiPSC-CV (control) or MS06. Monoclonal or polyclonal αSyn immunofluorescent staining in MS06 neurons compared with CV. Scale bar, 50 μm. (**D**) Western blot from total protein extract from CV and MS06 neuron homogenates. Mfn and Drp1 were quantified and normalized to tubulin as control (n = 3 independent experiments). (**E**) Representative images of neurons transfected with mito-GFP for mitochondrial morphology analysis of hiPSC-CV, MS06 clone, or MS06 plus A53T αSyn. Inset: magnification of axonal mitochondria. Scale bar, 30 μm, inset, 20 μm. (**F**) Representative images obtained from MS06 differentiated neurons showing abnormal elongated, aggregated, and interconnected axonal mitochondria. (**G**) Significant increase in mean mitochondrial size per axon was observed in MS06 neurons when compared to CV-derived neurons, while a reduction in size was observed upon A53T αSyn overexpression in MS06 neurons. Data is represented as mean ± SEM (n = 25 axons per condition from 2 independent experiments). One-way ANOVA followed by Dunnett’s post-test, *p < 0.05. Pooled mitochondria probability density by size distribution plots between MS06 and CV or A53T αSyn transfected MS06 and CV. Kolmogorov-Smirnov test performed for each comparison, ***p < 0.001, ****p < 0.0001 (n > 280 mitochondria per condition from 2 independent experiments).

To test whether the αSyn N-terminal domain controls the shape and morphology of axonal mitochondria, we disrupted the integrity of the α-helix structure in hiPSC. Directed CRISP-R/Cas9 genome editing of Craig Venter’s hiPSC line (CV) was performed to generate isogenic lines with disruptions in αSyn conformation using a specific short guide RNA directed to the exon 2 sequence^39^ (Fig. 5A). A PCR strategy using αSyn exon 2-specific primers was used to screen for positive clones with genomic insertions (Fig. 5A). One putative hiPSC-CV CRISPR/Cas9-modified clone (MS06) showed two modified alleles, one with a nucleotide insertion that shifted the reading frame, and the other with a net 18-amino acid insertion maintaining the downstream frame and giving rise to a higher molecular weight αSyn (Figure S7). αSyn expression in human-derived neurons and neural stem cells (NSC) from the MS06 clone revealed an increase of 2 kDa in αSyn molecular weight compared with isogenic controls or mouse hippocampal neurons (Fig. 5B). This confirmed the stable modification of αSyn protein with an insertion disrupting the proximity of the two α-helix N-terminal domains but with intact downstream sequence. Neuronal differentiation, polarity acquisition, and αSyn expression levels were tested in neurons differentiated from clone MS06 and compared with control hiPSC-CV derived neurons (Fig. 5C). After 14 days in culture, control and CRISP-R/Cas9-modified MS06 neurons were transfected with mito-GFP to assess mitochondrial morphology. MS06 neurons were found to display elongated morphologies of axonal mitochondria (Fig. 5E). Quantification of axonal mitochondria showed a significant 35% increase in average mitochondrial length with an abnormal distribution in MS06 neurons compared with control hiPSC-CV neurons (Fig. 5G). In addition, MS06 neurons contained abnormal mitochondrial morphologies, such as branched and interconnected mitochondria (Fig. 5F). To test whether these changes in mitochondrial morphology were dependent on a physiological role for αSyn in mitochondrial homeostasis, we overexpressed A53T αSyn in MS06 neurons. Interestingly, A53T αSyn overexpression led to significant reductions in mitochondrial length and distribution in MS06 neurons that resulted in even smaller mitochondria than in control hiPSC-CV neurons (Fig. 5E,G). To determine whether disruption of αSyn resulted in modification of fusion/fission-related protein levels, e.g., mitofusin (MFN) and Dynamin related protein 1 (DRP1), we performed Western blot analysis of CV control or MS06 neurons. Quantification of protein levels revealed similar amounts of MFN and DRP1 expression in MS06 and control neurons (Fig. 5D), suggesting that mitochondrial elongation in MS06 neurons is promoted without changes in expression of mitochondrial quality control proteins. However, this experiment did not rule out whether MFN or DRP1 activity may be driving the fragmentation phenotype. Together, these results strongly support a physiological role for αSyn in the control of mitochondrial morphology, which could be linked to a direct interaction of the N-terminal domain with the mitochondrial membrane.

## Discussion

Using a relevant human neuronal cell model, we provide novel evidence supporting the following conclusions: (i) αSyn variants have differential effects on mitochondrial transport and size, which is linked to levels of expression and mitochondrial localization; (ii) αSyn-dependent mitochondrial fragmentation requires direct interaction with the OMM; (iii) disruption of the N-terminal domain induces changes in mitochondrial morphology, supporting a physiological role for αSyn in mitochondrial size regulation; and (iv) this mechanism is not restricted to human-derived neuronal cells.

The role of αSyn in mitochondrial function has been the subject of extensive research in the context of neuronal pathology^40^. Mitochondrial fragmentation has been described in several PD models using different αSyn variants, although the specific effect of the different alleles has not been consistent^29–31, 41–43^. Comprehensive studies in relevant human cellular models to test the effect of αSyn mutation, dosage, and localization on mitochondrial fragmentation have been lacking. Here, we used human-derived neurons to characterize the effect of overexpression of three αSyn variants on axonal mitochondria. A53T αSyn overexpression led to significant reductions in axonal mitochondrial size. Milder size reductions were observed upon WT αSyn overexpression, but not in A30P overexpression. These differential αSyn effects on mitochondrial size were also dose dependent. The significant effect on mitochondrial size observed upon A53T overexpression suggests a gain-of-function mechanism, while the lack of effect of A30P, even upon high expression levels, suggests a loss of function. To determine whether the observed differences in mitochondrial size were related to a direct association of αSyn with the mitochondria, we performed subcellular fractionations in αSyn-overexpressing neuroblastoma cells. A53T αSyn localization within the mitochondrial fraction was enhanced compared with WT αSyn, while A30P αSyn showed reduced mitochondrial localization. These results suggest that αSyn might exert a functional regulation of mitochondrial morphology that can be modulated by different mutations.

The intrinsic biophysical properties of αSyn allow interaction with lipids, mediating a physiological function in synaptic vesicle docking to the plasma membrane^44, 45^. αSyn association with mitochondria has been proposed to depend on the helical conformation of the N-terminal domain of αSyn^46^. Disease-associated mutations in αSyn, which are all located within this domain, impact αSyn membrane binding affinity, with the A53T mutation having the highest membrane affinity and A30P having a lower membrane affinity compared with WT αSyn^22^. These differential properties correlate with our results on mitochondrial size and with reports suggesting mutation-dependent effects on mitochondrial fragmentation and defects in import machinery^47^. The association of αSyn with the OMM has been initially reported in mouse neurons^48^. This association seems to be increased under artificially-induced low intracellular pH conditions in cell lines overexpressing αSyn variants^49^. To determine whether a specific mitochondrial interaction mediated fragmentation, we utilized a dimerization system to target the different αSyn variants to the OMM, while maintaining availability of the N-terminal domain to interact freely with membrane molecules. Interestingly, WT and A53T αSyn delivery to the OMM resulted in a rapid and dose-dependent mitochondrial fragmentation phenotype, more pronounced than by their overexpression alone. In contrast, A30P did not alter mitochondrial size, even at high concentrations of dimerizing agent. Our results with A30P are consistent with previous reports that indicate an inability to induce fragmentation, little impact on mitochondrial import machinery, and decreased induction of micelle fusion *in vitro* ^29, 50^. A relationship between membrane curvature and rates of fusion is thought to occur in mitochondria as a result of changing lipid and protein composition^50, 51^. When overexpressed, the enhanced association of αSyn to the OMM either by protein-protein or protein-lipid interaction could lead to a decrease in fusion rate^29^. Consequently, mitochondrial fragmentation is favored leading in some cases to mitophagy^52, 53^. Taken together, these results strongly suggest that the effect of αSyn on fragmentation depends on a direct association with the mitochondria.

Given the enriched expression of αSyn in neuronal cells, many endogenous roles have been proposed, including control of synaptic vesicle release^54^, modulation of secretory pathways^55^, and even vesicle transport regulation^37^. However, whether endogenous αSyn exerts a physiological role in neuronal mitochondria quality control has remained unclear. Some clues arise from αSyn knock-out mice, which display a higher resistance to mitochondrial neurotoxins used to model PD^56^. A previous observation that αSyn knockdown suppresses stress-induced mitochondrial fission phenotypes also suggests a physiological regulation of mitochondrial dynamics^9^. In addition, the N-terminal domain of αSyn has been implicated in the control of mitochondrial fragmentation^31, 46^. The morphology of mitochondria also depends on recovery of membrane-enriched fusion-fission proteins found in mitochondria-associated ER membranes (MAM)^57^. When overexpressed, α-Syn modulates the mitochondria-MAM interaction affecting the transfer of calcium from ER to mitochondria in cell lines^58, 59^. Pathogenic mutations in α-Syn when localized to MAMs reduced the mitochondria-ER interaction while induced mitochondrial fragmentation^42^. The mechanism exerted by α-Syn in MAMs hint at a possible, as yet unexplored compartment that might be relevant for understanding the role of α-Syn in PD pathogenesis. To further test the hypothesis that α-Syn N-terminal domain interact with membranes to regulate the shape of mitochondria, we performed CRISP-R/Cas9-mediated genome editing to disrupt the proximity of the α-helix domains of endogenous αSyn in hiPSC. A shift in mitochondrial morphology towards elongated sizes and branched mitochondria was observed in axonal projections of CRISP-R/Cas9-modified human-derived neurons, which was not dependent on changes in MFN or DRP1 proteins levels. In addition, the strong fragmentation elicited by A53T overexpression in these modified cells supports a physiological role for the N-terminal domain of αSyn in the regulation of mitochondrial dynamics.

The association of αSyn with molecular motors^60, 61^, and the accumulation of αSyn aggregation bodies suggest that αSyn plays a central role in the induction of axonal transport defects^36, 62^. Consistent with this view, we showed that overexpression of WT or mutated forms of αSyn in human-derived neurons resulted in significant defects in anterograde/retrograde mitochondrial flux associated with abnormal mitochondrial distribution in axons. Similar dynamic defects have been reported in cell lines and mouse neurons overexpressing WT or A53T αSyn overexpression; A30P was not analyzed^30^. Axonal mitochondrial mobility drives the fusion-fission rates that control mitochondria morphology^63^, and the same counterbalance regulation of mitochondrial transport has been suggested when fusion-fission is impaired^64^. This crosstalk between fusion-fission and axonal flux is further influenced by mitochondrial membrane potential, which acts as a sensor of mitochondrial integrity and favors retrograde over anterograde transport^65^. The axonal transport defects observed upon αSyn overexpression are consistent with the fragmentation phenotype we observed; however, mitochondrial membrane potential remained unchanged at the time of analysis. This opens the door to further investigation into the effects of long-term αSyn overexpression on mitochondrial transport and function.

Many genes associated with PD converge on the mitochondria as a crucial etiological factor in disease initiation. PINK1, Parkin, and DJ-1 interact within similar pathways that regulate mitophagy, transport, and mitochondrial fusion^13, 16, 17^. LRRK and VPS35 proteins control mitochondrial morphology by interacting with the fission protein DLP1, leading to an increase in mitochondrial fission^12, 15^. Our findings provide further evidence to suggest a physiological role for αSyn in the control of axonal mitochondrial transport and morphology in human-derived neurons, which can be modulated by mutation or gene dosage. The physiological and pathological role of αSyn in mitochondrial homeostasis uncovered here may lead to a common pathway underlying PD pathogenesis, which is ultimately relevant for understanding neurodegenerative diseases and for the future design of preventive or therapeutic strategies for PD.

## Methods

Detailed methods can be found in the SI appendix.

### Neural differentiation

The human embryonic stem cell (hESC) line Hues9 (H9, WiCell Research Institute) and induced pluripotent stem cell line (Craig Venter: CV iPSC SD2010–125, UCSD) were differentiated into polarized and functional neurons following established protocols^32^ as described in the Supplementary Experimental Procedures. pLv-Bobi-CMV-αSyn (WT, A30P, A53T) vectors were introduced into differentiated neurons using either lipofectamine transfection or viral transduction at DIV14 for live or fixed experiments.

### Live imaging of fluorescent mitochondria

hESC- or hiPSC-derived neurons were live-imaged in CO_2_ and temperature control for fluorescent axonal mitochondria visualization and dynamic tracking of mitochondrial movement and morphology as previously described^18, 34^. Acquisition details and analyses are described in the Supplementary Experimental Procedures.

### αSyn targeting to mitochondria

WT, A30P, and A53T αSyn-FRB vectors were co-transfected into human neurons with GFP-FKBP(x2)-ActA to drive mitochondrial targeting of αSyn in the presence of 200 or 500 nM rapalog dimerizing agent for 6 h. Transfected and treated neurons were rinsed, fixed, and immunostained prior to analysis of mitochondrial morphology.

### αSyn N-terminal domain disruption by Crisp-R/Cas9 genomic editing

CrispR/Cas9 disruption of the N-terminal domain of αSyn was performed using a guide RNA targeting the SNCA exon 2 sequence between both alpha helices of the N-terminal domain of αSyn in iPSCs (CV iPSC). Each clone was tested for insertions or deletions by PCR amplification with specific primers flanking the genomic site of the gRNA target. Clone MS06 was sequenced and selected for analysis of neural differentiation and mitochondrial morphology.

## Electronic supplementary material

Supplemantal Information

Movie 1

Movie 2

Movie 3

Movie 4
