# Supplementary material for: αSynuclein control of mitochondrial homeostasis in human-derived neurons is disrupted by mutations associated with Parkinson’s disease
Source: Sci Rep. 2017 Jul 11;7:5042. doi: 10.1038/s41598-017-05334-9 (PMC5506004; doi:10.1038/s41598-017-05334-9)
Supplement: Supplementary file 1 — Supplemantal Information [file 41598_2017_5334_MOESM1_ESM.pdf]

αSynuclein control of mitochondrial homeostasis in human-derived neurons is disrupted by mutations associated with Parkinson's Disease

Victorio Martin Pozo Devoto[1, 2], Nicolas Dimopoulos[3], Matías Alloatti[1], María Belén Pardi[5], Trinidad M. Saez[1,4], María Gabriela Otero[1], Lucas Eneas Cromberg[1], Antonia Marín-Burgin[5], Maria Elida Scassa[3], Gorazd B. Stokin[2], Alejandro F. Schinder[6], Gustavo Sevlever[3] and Tomás Luis Falzone[1, 4 *].

[1] Instituto de Biología Celular y Neurociencias, IBCN (UBA-CONICET), Facultad de Medicina, Universidad de Buenos Aires. Paraguay 2155, Buenos Aires, CP1121, Argentina.
[2] Center for Translational Medicine, International Clinical Research Center, St. Anne's University Hospital Brno, Brno, Czech Republic.
[3] Instituto FLENI
[4] Instituto de Biología y Medicina Experimental, IBYME (CONICET). Vuelta de obligado 2490, Buenos Aires, CP 1428, Argentina.
[5] Instituto de Investigación en Biomedicina de Buenos Aires (IBioBA) –CONICET - Partner Institute of the Max Planck Society, Buenos Aires, Argentina.
[6] Laboratorio de Plasticidad Neuronal, Fundación Instituto Leloir (IIBBA – CONICET), Av. Patricias Argentinas 435, Buenos Aires, CP C1405BWE, Argentina.

[*] Correspondence: tfalzone@fmed.uba.ar

Supplemental Experimental Procedures

Cell Culture

Culture of hPSCs and neuronal differentiation

Human embryonic stem cell (hESC) line Hues9 (H9, WiCell Research Institute) and induced pluripotent stem cells line Craig Venter (CV iPSC SD2010-125, UCSD), were obtained under material transfer agreement to UBA, Tomas Falzone. hiPSC GM25430 purchased from Coriell institute for medical research. Both hESC and hiPSC colonies were maintained in p100 adherent culture plates (Corning) with a feeder layer of irradiated E13 mouse embryonic fibroblast (iMEF) at 37°C, 5%CO_2, cells were split weekly 1:3. Expansion medium was comprised of Dulbecco's Modified Eagle's KO Medium (KO-DMEM) supplemented with 10% Knockout Serum Replacement (KSR), 2 mM non-essential amino acids, 2 mM L-glutamine, 100 U/ml penicillin, 50 µg/ml streptomycin, 0.1 mM β-mercaptoethanol and 4 ng/ml of bFGF. All reagents were obtained from Life Technologies (Carlsbad, CA, USA). Efficient neural derivation of the pluripotent cells was achieved by adaptation of a previously published protocol (Figure S1) (28). As a brief summary of the procedure: Pluripotent colonies were enzymatically de-attached from its feeder layer using Colagenase IV (1mg/ml) (Gibco) for 25 min at 37°C. Cell aggregates obtained from 2 p100 culture plates, were then suspended in 25 ml of expansion medium (without bFGF) in a T75 culture flask and kept in flotation for a total of 4 days at 37°C 5% CO_2, changing half of medium volume every other day. During this period, the cell aggregates acquire a spherical shape. The fifth day, medium was replaced for 25 ml Neural Induction Medium (NIM), comprised by: DMEM/F12 supplemented with, non-essential amino acids (1:100, Invitrogen), N2 supplements (1:100, Gibco), and 1 mg/ml Heparin at 37°C 5% CO_2. After 2 days the cell aggregates were attached in laminin pre-coated (20µg/ml) 6-well plate. About 30-40 aggregates per well were then kept in 2 ml of NIM at 37°C 5%CO_2 changing most of the medium every other day for 9 days.

On the attachment stage, formation and maturation of neural tube-like structures and neural progenitors enriched rossettes was observed. On the 9th day, those multi-cell layered structures were mechanically dissected. These aggregates of neural rosettes cells were then transferred to T25 flasks with 15 ml of NIM supplemented with B27 supplement (1:50,Gibco) changing medium every other day. After 12 days in suspension, the neural progenitors enriched spheres were enzymatically digested with 500 ul of trypsin-EDTA [0,25%] and Accutase (1:1) for 3 min at 37°C. The reaction was ended by the addition of 500ul of Soybean Tripsin inhibitor (0.5mg/ml) for 3 min at 37°C. cells were then resuspended in neuronal differentiation medium (NDM) comprised of: Neurobasal medium supplemented with N2 supplement (1:100,Gibco, 17502-048), B27 supplements (1:50,Gibco, 17504-044), laminin 1 µg/ml (Invitrogen), cAMP 1uM (1:10000, Sigma), ascorbic acid 200ug/mL (1:1000, Sigma), BDNF 100 ug/ml (1:10000, R&D), GDNF 100 ug/ml (1:10000, R&D). After mechanically disaggregation of the spheres using a p1000, cells were centrifuged for 2 min at room temperature, attached to laminin 20ug/ml pre coated 24-well plate in 600 ul NDM. Changing half of the volume in each well every other day for at least 14 days until terminally differentiated.

Neural Stem Cells cultures

Neural Stem Cells (Gibco, H9 hESC derived) were plated on 24 MW dishes on matrigel coated coverslips and mantained in growth media: KO DMEM/F12, StemPro Neural supplement 2% (gibco), Glutamax, bFGF and EGF (both 20ng/ml). Media change was performed every other day.

Mouse primary Hippocampal neuronal cultures

C57/BL6 mice were used to generate primary hippocampal cultures. The mice were housed in temperature and light/dark-controlled rooms and the procedure were approved under university protocols (UBA CICUAL-456/2010). Briefly, newborn hippocampal brain regions from C57/BL6

mice were dissected on postnatal day 1. Hippocampi were incubated in a 0.22-mm-filtered mixture of 45 U of papain in Phospate-Buffered Saline (PBS) enriched with 0.05% of DNase for 20 minutes at 37°C. After, gentle mechanical dissociation cells were then transferred to poly-D-lysine-coated coverslips and covered with Neurobasal medium supplemented with L-glutamine and B27 (Life Technologies), and cultured at 37°C 5%CO_2.

Human neuroblastoma cell line culture

SHSY5Y (ATCC SHSY5Y) were maintained in p100 dishes with growth medium at 37°C 5%CO_2 and split once per week 1:5. Growth medium composition: 250 ml DMEM F12, 10% fetal bovine serum (Gibco), glutamax and Pen/Strep (Life technologies).

Electrophisiology

For electrophysiological recordings, cultured cells were perfused in (mM): 125 NaCl, 3KCl, 3 $CaCl_2$, 2 $MgCl_2$, 5 HEPES, 5 HEPES Na, dextrose; 315 mOsm and bubbled with 95% O_2/5% CO_2. Whole-cell recordings were performed using microelectrodes (5-6 MΩ) filled with (in mM): 150 potassium gluconate, 4 $MgCl_2$, 10 HEPES buffer, 0.1 EGTA, 1 NaCl, 4 ATP-tris, 0.3 GTP-tris, 10 phosphocreatine and 5 ug/ml alexafluor 488 (Invitrogen); pH=7.3; 290 mOsm. Spiking was assessed keeping the resting membrane potential at −60 mV in current clamp and passing successive depolarizing current steps of 10 pA increment and 500 ms duration. Voltage-dependent Na^+ and K^+ currents were measured in voltage clamp after leak subtraction using a p/-6 protocol and detection of the fast inward peak for Na and the late outward plateau for K. Series resistance was typically 10–20 MΩ, and experiments were discarded if higher than 30 MΩ. Recorded neurons were visually identified by fluorescence and infrared DIC videomicroscopy. Recordings were obtained using Axopatch 200B amplifier (Molecular Devices), digitized (Digidata 1322A; Molecular Devices), and acquired at 20 KHz onto a personal computer using the pClamp9 software.

Immunofluorescence Staining

hESCs, differentiated neurons, NSCs and hippocampal neurons were analyzed for immunofluorescence. Briefly, the cells were rinsed with ice-cold PBS and fixed in PFA 4% + Sucrose 4% in PBS for 30 min. After two washes cells were permeabilized and blocked with 0.1% Triton X-100 in PBS with 10% normal goat serum for 60 min, and stained with the corresponding primary antibodies over night: anti-Oct-3/4 (mouse, clone C-10 SC), anti Pan-Ncam (mouse, 556324, BD Pharmigen), anti-Oligodendrocyte Marker 4 (mouse, MAB 345, Millipore), anti-Tuj1 (mouse, AB78078, Abcam), anti-Nanog (rabbit, clone H-155 SC), anti-Nestin (rabbit, Clone AB5922 Chemicon), anti-TH (Clone P40101, Peel Freez), anti-Pax6 (Ab 5790, Abcam), anti-Gfap (AB5804, Chemicon), αSyn-mab (619786, BD-Biosciences), αSyn-pab (AB5038, Chemicon), Tau (T6402, Sigma), pTau (CP13, Peter Davies), GFP (A11122, Invitrogen), Sox2 (S9072, Sigma), COX-I (A6405 Invitrogen). After three rinses, fluorescent secondary antibodies incubation for two hours: Alexa Fluor 568 conjugated with anti-mouse or anti rabbit IgG and Alexa Fluor488 conjugated anti-rabbit or anti-mouse IgG from Molecular Probes/Invitrogen were used to localize the antigen/primary antibody complexes. The cells were counterstained with 4-6-diamidino-2-phenylindole (DAPI) (Molecular Probes/Invitrogen, Carlsbad, CA, USA). The images were acquired either with a Nikon DXN1200F digital camera, which was controlled by the EclipseNet software (version 1.20.0 build 61) or the OlimpusIX71/IX81 Inverted Microscope and its control software IX2-BSW.

RNA Isolation, RT-PCR and RT-qPCR

Total RNA was extracted from hESCs using Trizol (Invitrogen, Carlsbad, CA, USA) as recommended by the manufacturer. cDNA was synthesized from 1000 ng of total RNA using 15 mM of random hexamers (Invitrogen, Carlsbad, CA, USA) and MMLV reverse transcriptase (Promega, Madison, WI, USA), according to manufacturer's instructions. The cDNA samples were diluted fivefold. The SYBR_Green-ER™ qPCRSuperMix UDG (Invitrogen, Carlsbad, CA,

USA) was used for all reactions, following manufacturer instructions. The qPCR studies, PCR amplification and analysis were performed with the Step one Sequence Detector and its software (PE Applied Biosystems, Foster City, CA, USA). A melting curve analysis was performed immediately after amplification at a linear temperature transition rate of 0.3°C/s from 70 to 89°C with continuous fluorescence acquisition. Ribosomal protein 7 (Rpl7) was used as loading control, relative quantification was assessed by the ΔΔct method assuming an amplification efficiency of 100% ($2^{\Delta\Delta ct}$). All values were relativized to the undifferentiated state at day 1. qPCR Primers and Fragment size (5'-3'):

Oct4: Fwd: CTGGGTTGATCCTCGGACCT, Rev: CACAGAACTCATACGGCGGG;

Nanog: Fwd: AAAGAATCTTCACCTATGCC, Rev: GAAGGAAGAGGAGAGACAGT;

Pax6: Fwd: CACGTGTCCAACGATGTG, Rev: GTCGCTACTCTCCGTTTACTAC;

Nestin: Fwd: GGCGGTGGCTCCAAGACTTC, Rev: GGTAGCAGGCAAGGGTGAGG;

Tuj1: Fwd: TGGGCGACTCGGACTTGC, Rev: CCACTCTGACCAAAGATGAAATTG;

TH: Fwd: CGACCCTGACCTGGACTTGG, Rev: GGCGTGCGTGGCGTAGAG;

Rpl7: Fwd: AATGGCGAGGATGGCAAG, Rev: TGACGAAGGCGAAGAAGC.

<u>Transfection</u>

Plasmids used for transfection include the following constructs. plv vectors with CMV promoter containing WT, A30P or A53T αSyn inserts (kind donation from Dr. Eliezer Masliah, UCSD, San Diego). pcDNA3 vector containing EGFP, cherry, mito-GFP, or empty. pCI-Neo vectors expressing GFP-FKBP-ActA, WT, A30P or A53T αSyn-FRB fusion proteins.

Human neurons, NSCs, mouse hippocampal neurons or SHSY5Y line were transfected following the same protocol. Briefly, using lipofectamine 2000 a mix of reagent:DNA (2ul:1ug) was premade on OptiMEM. Culture media was replaced with OptiMEM 30 min before the incubation. Cultures were kept for two hours in incubator with the mix, after which it was aspirated and culture media restored. All analysis were performed at least 24 hs after the

transfection. Transfection efficiency in human-derived neurons was lower than 5% analyzed by imaging and flow cytometric analysis.

Time-lapse imaging

Mitochondrial movement in neurons was recorded following previous described protocols (30, 17) using an inverted epifluorescent microscope (Olympus IX81) connected to a CCD camera (Olympus DP71, 12.5 megapixels) with a 60X objective (1.40 NA). During time-lapse acquisition cultures were kept at 37°C and 5% CO2 using a heating stage and a CO2 chamber (Olympus). Time-lapse movies of 150 seconds (with 3 seconds intervals) were acquired from approximately 20 different axons in each well. Movies were transformed to kymographs with ImageJ (NHI) using the multiple kymograph plug-in, and movement parameters (eg. movement proportions, velocities, segmental velocities), size and densities were extracted for analysis.

Transduction

Cells were transduced with either lentiviral plv Bobi vector control or plv Bobi carrying a CMV promoter driving the expression of WT, A30P, or A53T αSyn (Dr. Eliezer Masliah, UCSD, San Diego) packaged in pseudolentiviral particles in co-transfected HEK-293T cells with plv Bobi vector together with vectors encoding packaging and envelope proteins (CMVΔ8.9 and CMV-VSVg, respectively), using Lipofectamine Plus reagent (Invitrogen). Two days after transfection, viral particles were harvested, treated with DNaseI and 2 mM $MgCl_2$, filtered through a 0.45 μm pore size filter and concentrated by ultracentrifugation at 100,000 g_{av} for 90 min. Titers of concentrated virus obtained were between $0.5\text{-}2\times10^9$ TU/ml.

Mitochondrial Membrane Potential

Neurons were differentiated in a 24 multi-well (corning). These terminally differentiated neurons were transduced with a MOI of 30 for each αSyn construct or empty vector as negative control.

7 days after transduction cultures were incubated with JC1-Dye (1µM) (Invitrogen™, Life technologies) in PBS at 37°C 5% CO_2, for 15 minutes. Mitochondrial ionophore, Carbonyl cyanide-4-(trifluoromethoxy)-phenylhydrazone (FCCP) (10μM), was used as a positive control and co-incubated with JC1. Excess dye was washed once with PBS. Medium was changed for NDM until visualization. Cells were observed in DMEM minus phenol red, using a heating stage and a CO2 chamber (Olympus). Images were acquired on two different channels: excitation (BP460-495) for green, and excitation (BP530-550) for red. Colocalization of both channels was assessed and Manders correlation coefficient was calculated using ImageJ JACOP plug-in, untreated control was used to establish the threshold for both red and green channels for each multiwell replicate. Quantification of signal was performed by adapting the Manders colocalization index: the value that results from the formula [1-Manders (Red or Green)] which gives a proportion of exclusively green (impaired) or red (healthy) pixels (mitochondria). Thus, the FCCP treatment shifts almost completely the proportion to exclusively green.

Subcellular Fractionation

To assess αsyn localization on SHSY5Y cells were transfected as detailed elsewhere with vectors containing either empty, WT, A30P or A53T αSyn. Cells were restored to DMEMc for 72 hs and subcellular fractionation was performed. Briefly, cells were washed once in cold PBS prior to resuspension in homogeneizing buffer H (10 mM Tris pH 7.4, 10 mM KCl, 2 mM EGTA, inhibitor cocktail, NaF and Na_3VO_4, 300 mM sucrose). Cells were harvested and mechanically treated with 30 strokes of a Teflon Dounce homogenizer. Total fraction was obtained, while the rest was centrifuged at 2000 g for 10 min. Pellet (nuclear fraction) was resuspended in Buffer H, and supernatant was centrifuged at 15000 g for 15 min. Mitochondrial fraction (obtained from the pellet) was resuspended in Buffer H, while remaining supernatant corresponds to the citosolic fraction. Fractions were quantified using the BCA method, cracked in sample buffer and frozen.

Western Blot

Protein was extracted from cells in lysis buffer (10 mM Tris-Hcl, PH 7.4, 0.8 M NaCl, 1mM EGTA, 0.1% triton X100, protease inhibitors) at 4°C. Protein concentration was measured by BCA assay (Bio-Rad). Equal protein amounts (20 µg) in 20 µl of loading buffer (0.5 % bromophemol blue; 10 % glycerol; 10% 2-Mercaptoethanol) were loaded onto 12% (w/v) SDS-polyacrylamide gels (30% Acrilamide and N,N`-Methylenebisacrilamide, SIGMA). Gels were blotted onto nitrocellulose membranes (Whatman, USA) using the wet transfer method (Tris-Glycine). Membranes were blocked in 5% (w/v) nonfat milk (Sancor) in 0.05% v/v Tween 20 in PBS (TBS-Tween) for 1 hour. Primary antibodies were incubated overnight at 4°C. VDAC, Tub, αSyn (mono y poly). Washes were performed 3 times in TBS-Tween and blots incubated with the appropriate secondary antibody conjugated to horse radish peroxidase (HRP) made in goat (Jakcson) for 2 hours at room temperature. Bands were developed using ECL incubations (Pierce) and exposed to films (Agfa) in cassettes for different times. Scanned images were analyzed using Image-J program.

FKBP/FRB heterodimerizing system

PCI-Neo vector with the insert GFP-FKBP(x2)-ActA and with the insert HA-BICD-FRB were kindly provided by Dr. Hoogenraad (Ultrecht University, Netherlands). WT, A30P and A53T αSyn were amplified by PCR from plv vectors with flanking primers introducing SalI and XbaI restriction sites. HA-BICD-FRB vector previously digested with SalI and XbaI was used for subcloning of HA-αSyn-FRB (WT, A30P and A53T). In-frame ligation and expression clones were selected by sequence analyses, western blot and immunofluorescent staining after transfection. Mitochondrial targeting of GFP-FKBP(x2)-ActA transfected neurons was assesed by incubation with the mitochondrial probe MitoTracker for 30 min (100 nM in NDM). After being rinsed, cells were visualized by fluorescence microscopy on NDM media. Rapalog incubation

was tested at different times and concentrations without toxicity up to 500 nM. αSyn-FRB localization to mitochondria was observed as short as after 120 min of incubation. αSyn-FRB on mitochondrial morphology was analyzed after 6 hrs of rapalog incubation following cell rinsing, fixing and antibody staining against αSyn and GFP.

Crispr/Cas9 genome edition

SNCA exon 2 genomic edition to disrupt the N-Terminal domain of αSyn was performed following CrispR/Cas9 specific DNA cutting strategy and NHEJ repair. Briefly, a guide RNA for targeting a sequence between both alpha helix of the N-terminal domain of αSyn was generated using the CrispR Design Tool from Zhang Lab web tool. This sequence of 19 nucleotides GGGTGTTCTCTATGTAGGT without the PAM sequence was included in a template backbone consisting of gRNA that presents a U6 promoter, a gRNA scaffold and a termination signal. This whole sequence was synthesized as 450 pb of dsDNA and cloned in a plasmidic vector pBlueScript. Control of site specificity for gRNA sequence was performed by the surveyor assay on HEKs cells. For expression of the nuclease Cas9 and selecting positive transfected cells a pCAG Vector with the expression of Cas9-2A-GFP was used. iPSCs (CV iPSC SD2010-125, UCSD) (on passage number less than 40) were lifted with accutase and electroporated with the Cas9-2A-GFP and the gRNA vectors, using Nucleofector kit for human stem cells with a Amaxa Nucleofector system. After electroporation, cells were plated to allow Cas9-2A-GFP expression. Cells were lifted with accutase and sorted for positive green signal, plating them in a concentration of 1000 cells per p100 dishes. Growing colonies where lifted manually and seeded individually in 96 well plates. Each clone was tested for ins/del by PCR amplification with specific primers flanking the genomic site of gRNA target. Different clones showing size changes were chosen for sequencing. MS06 clone was selected for having putative changes in both alleles. Differentiation protocol to obtain NSCs and terminally differentiated neurons was followed as previously detailed for western blots and mitochondrial morphology analyses.

Image analysis and statistics

Image analysis of mitochondrial size, intensity of cellular fluorescent signal and western blots were performed using ImageJ software. MultiKymograph plugin was used to generate kymographs from time-lapse movies, JACoP plugin for colocalization analysis and 3D Object Counter plugin for 3D mitochondria measurements. Statistical analysis and plotting was performed using Prism 6 (GraphPad Software) and Mathematica 9. *t*-test was used for comparison of two sample with Gaussian distribution. One-way ANOVA followed by Dunnet post-test. Two-way ANOVA followed by a Sidak's corrected comparison of Manders coefficient. Non parametric test were used in the case were normality was not achieved following Mann-Whitney U test or Kruskal-Wallis. Kolmogorov-Smirnoff was used for probability density distribution analyses.

Supplementary figures and legends

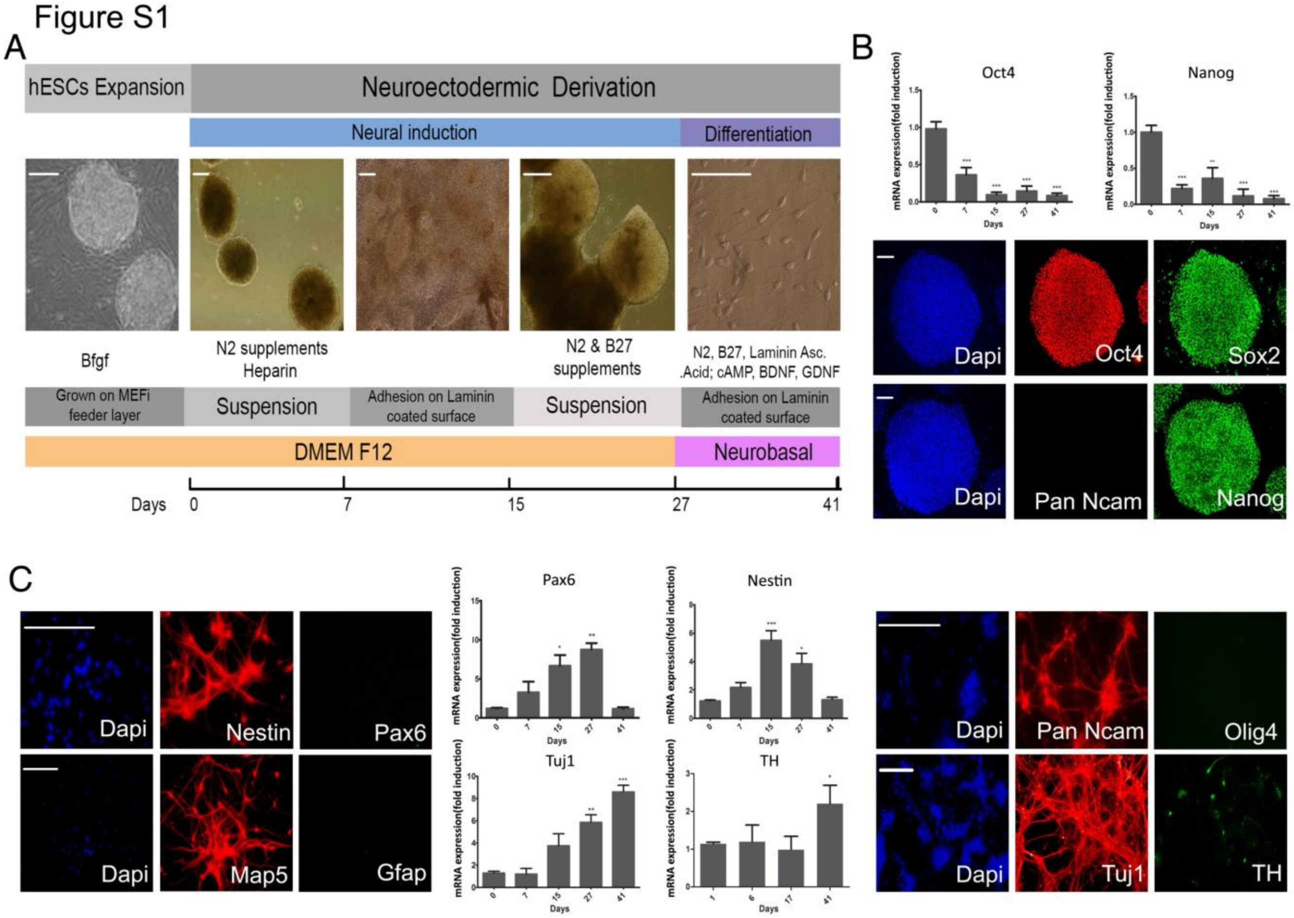

Fig.S1. Neuronal differentiation protocol from HUES9 hESCs. Related to Figure 1. (A) Scheme summarizing the implemented differentiation protocol (Zhang et al, 2010), brightfield images represent cell morphology in each stage of the process. (B) Transcriptional profile for pluripotency markers Oct4 and Nanog assessed by qPCR throughout the differentiation, data represented as mean ± SEM, n=4, one-way ANOVA ($p<0.05$) followed by a Dunnet post-test was performed. Immunofluorescence showing the presence of pluripotency markers Oct4, Nanog, Sox2 and absence of intermediate neuroectodermal marker Pan-Ncam in undifferentiated colonies expanded on irradiated mouse embryonic fibroblast feeder layer. (C) Left and right panels: immunofluorescence of terminally differentiated cultures (DIV14 on

terminal differentiation media, DIV41 of differentiation protocol) showing the presence of late neural markers Tuj1, Map5, TH; intermediate neuroectodermal markers Nestin and Pan-Ncam, and abscence of glial markers GFAP, Olig4 and early neuroectodermal marker Pax6; Center panel: mRNA expression assessed by qPCR of Pax6, Nestin, Tuj1 and TH along the differentiation process. Data represented as mean ± SEM, n=4, one-way ANOVA ($p<0.05$) followed by a Dunnet post-test was performed. ***$p<0.001$, **$p<0.01$, *$p<0.05$.

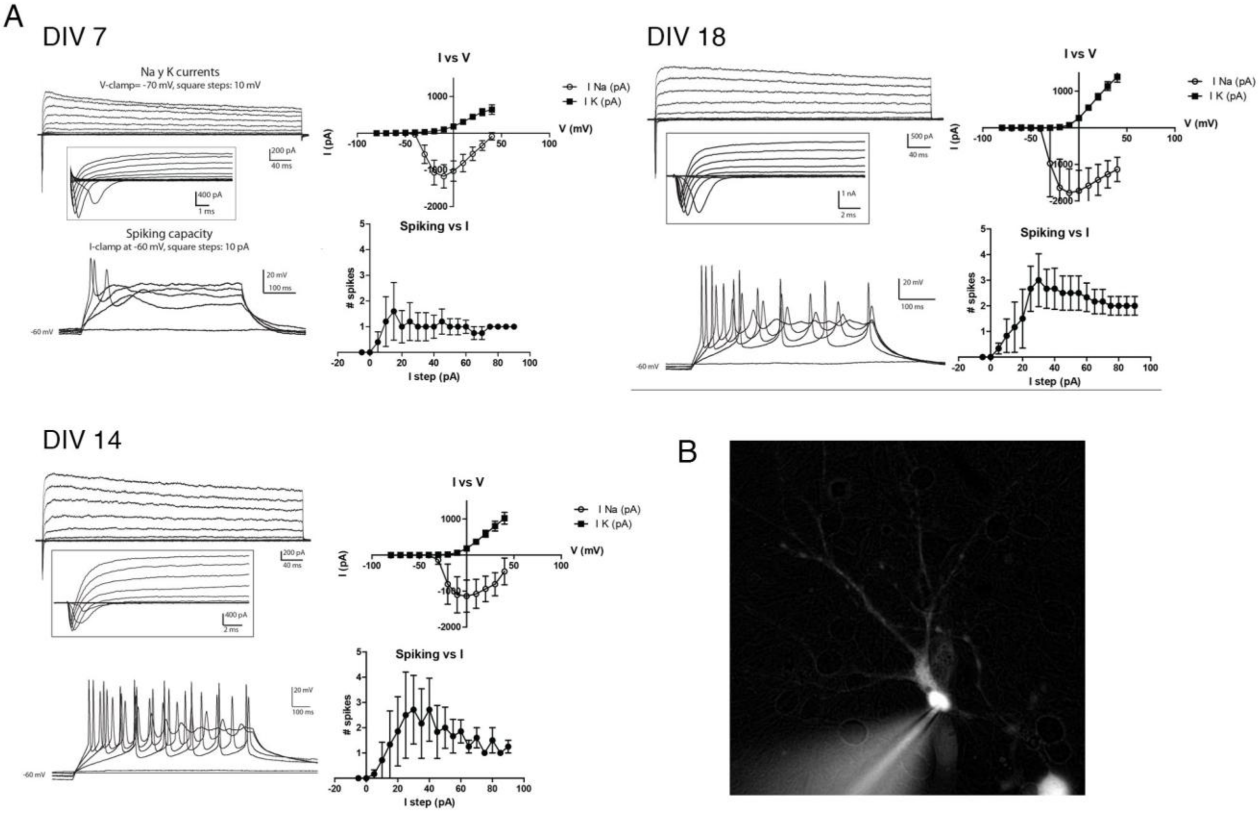

Fig.S2. Electrophysiological maturation of terminally differentiated neurons. Related to Figure 1. (A) Electrophysiological characterization of neuronal cultures in differentiation media DIV7, 14 and 18. Na^+/K^+ currents (voltage clamp: -70mV, square steps: 10 mV) typical traces and I (pA) vs V (mV) plot. Evoked action potentials (current clamp: -60 mV, square steps: 10 pA) typical traces and number of spikes vs. current step plots. Data in plots is represented as mean ± SEM of at least n=10 cells. (B) Micrograph showing a whole cell patch on DIV14 neuron, stained with alexa green.

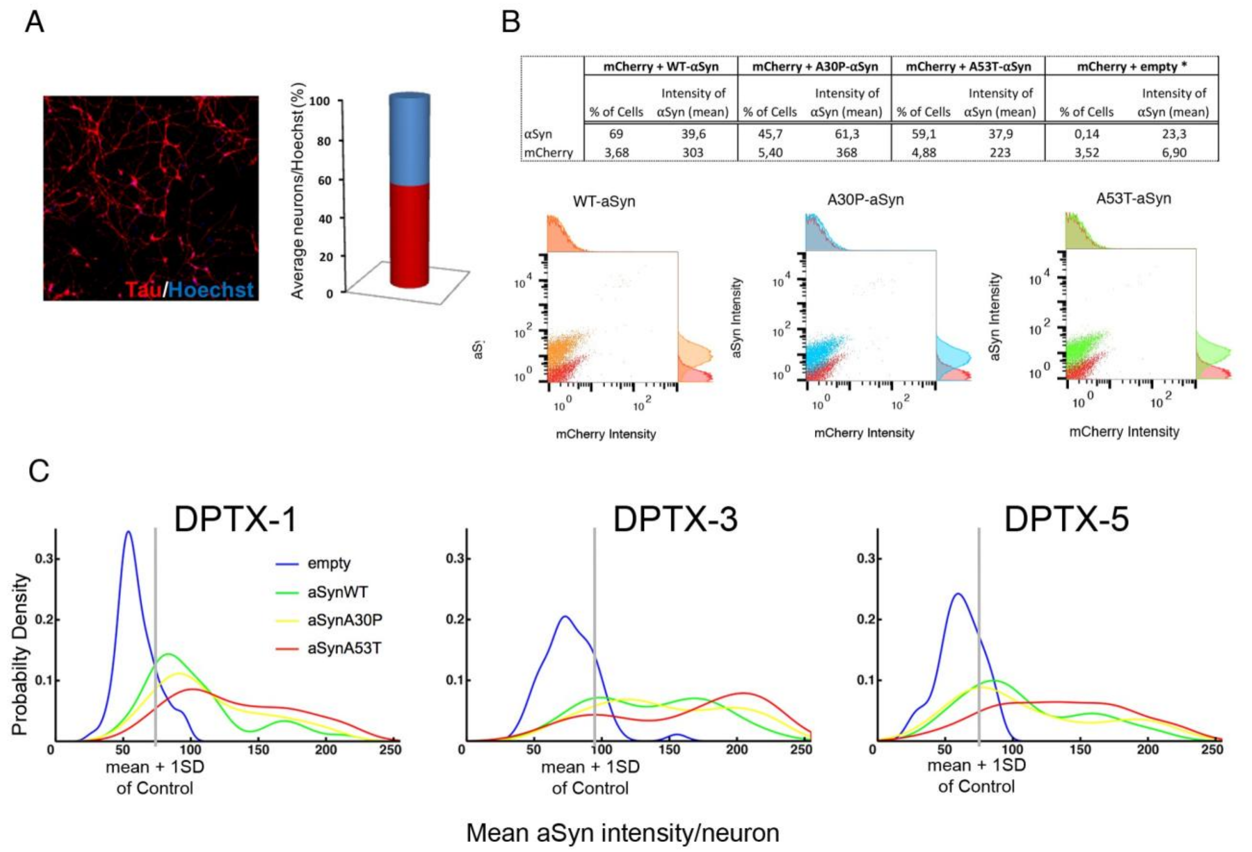

	mCherry + WT-αSyn		mCherry + A30P-αSyn		mCherry + A53T-αSyn		mCherry + empty *	
	% of Cells	Intensity of αSyn (mean)	% of Cells	Intensity of αSyn (mean)	% of Cells	Intensity of αSyn (mean)	% of Cells	Intensity of αSyn (mean)
αSyn	69	39,6	45,7	61,3	59,1	37,9	0,14	23,3
mCherry	3,68	303	5,40	368	4,88	223	3,52	6,90

Fig.S3. Expression levels of αSyn variants after transfection in stem cell derived neuronal cultures. Related to Figure 1. A). 21 days-old human-derived neurons in culture stained against tau antibody (red) and double stained for nucleus with Hoechst (blue). Percentage of neurons (red) from total identified by tau and Hoechst with clear neuronal morphology plotted against only Hoechst (blue). Neurons equal 55.17 % +/- 2.2 quantified from low magnification fields from 3 independent differentiation procedures. (B) Flow cytometry analysis showing the average αSyn intensity for co-transfected neurons with mCherry plus WT, A30P and A53T (red channel) compared with non transfected cells. Dot plots from cytometryc analysis are provided. (C) Neurons were co-transfected with EGFP and WT, A30P or A53T αSyn, and fixed after 1, 3 or 5 days post-transfection (DPTX). An immunofluorescence against αSyn was performed and only αSyn mean intensity fluorescence from EGFP expressing neurons was quantified. To set a

basal level of αSyn overexpression, a threshold line was arbitrary defined as the intensity equal to the mean + one standard deviation from the control treatment intensity distribution.

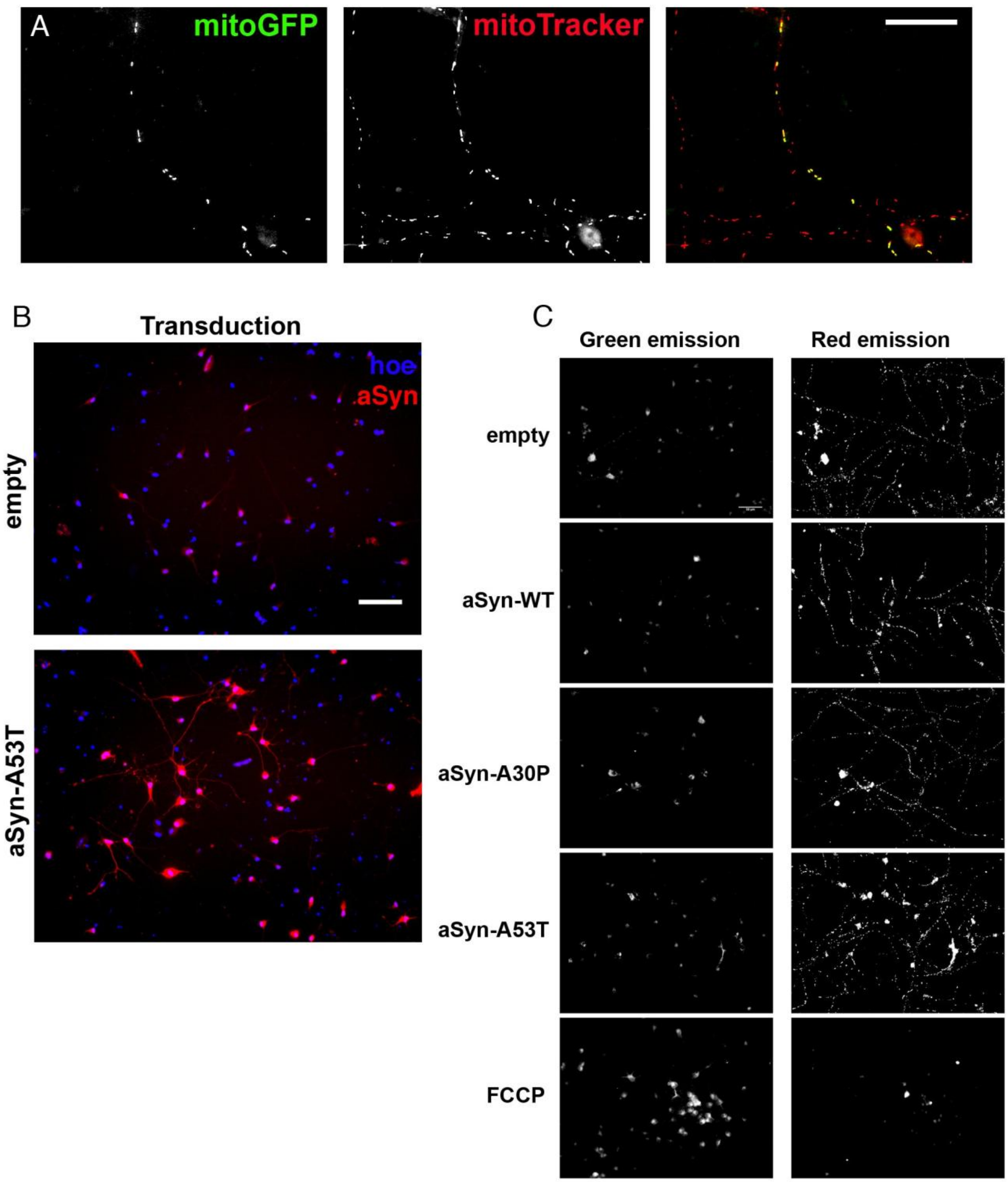

Figure S4. αSyn transduction and mitochondrial membrane potential analysis. Related to Figure 2. (A) Image from a live axonal projection in a neuron transfected with mito-GFP and stained with mitotracker red. Note the colocalization of the mitochondrial staining in yellow in merged image. (B) Immunofluorescence against αSyn reveals higher and ubiquous αSyn expression in

the αSyn transducted cultures. Scale bar: 50μm. (C) Micrographs showing independent emission of JC1 at 510-540 nm (green) and 575`LP (red) for empty, aSyn-WT, A30P, A53T or FCCP treated cultures. Scale bar: 50μm.

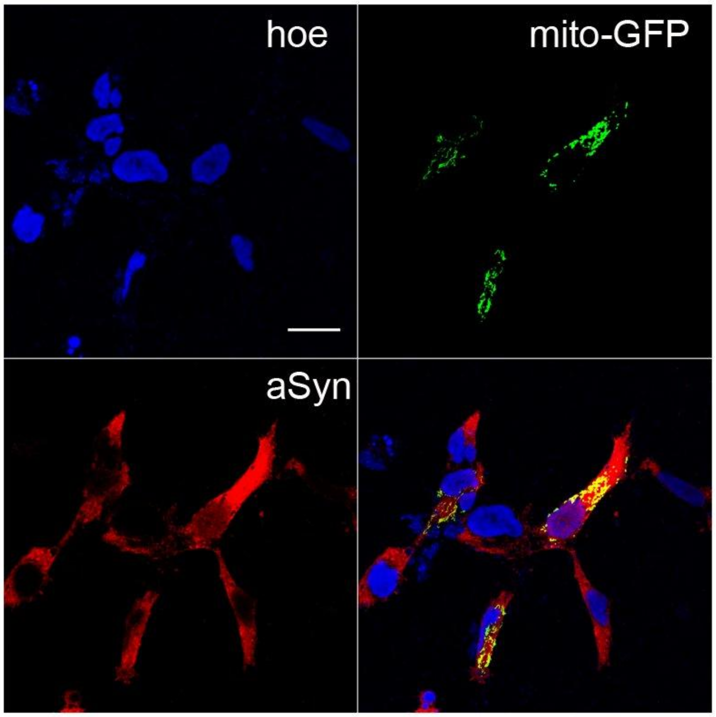

Fig.S5. Human NSCs mitochondrial changes induced by αSyn A53T overexpression. Related to Figure 3. (A) Micrographs showing αSyn immunostaining of NSCs overexpressing αSyn A53T. Overexpression conditions were the same as the ones detailed in Fig. 3G. Scale bar: 10μm.

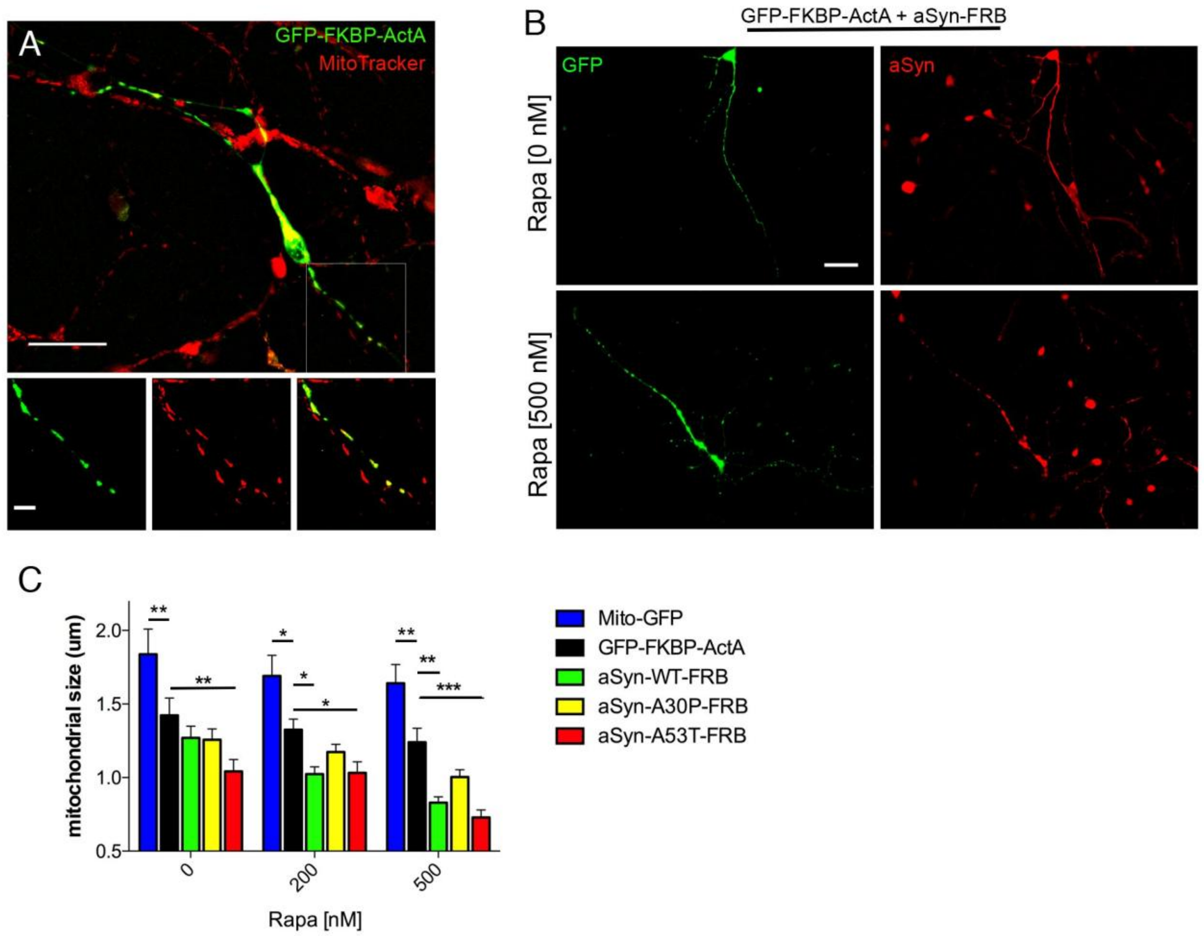

Fig.S6. GFP-FKBP-ActA and αSyn-FRB dimerization. Related to Figure 4. (A) GFP-FKBP-ActA is correctly delivered to mitochondria. GFP-FKBP-ActA transfected neurons were incubated with mitochondrial probe MitoTracker (Red). Micrographs showing colocalization are shown. Scale Bar upper panel: 50μm, lower: 10μm. (B) Low magnification micrographs of cultures co-transfected with GFP-FKBP-ActA and αSyn-FRB with or without incubation with Rapalog (Scale bar: 30μm). (C) Mean mitochondrial size per axon for neuronal cultures transfected with mito-GFP or GFP-FKBP-ActA plus empty, WT, A30P or A53T αSyn. Rapalog incubation (0, 200 or 500 nM) was performed for each condition. Data is represented as mean ± SEM (n=15). A two way ANOVA followed by a Dunnet post-test was performed, *p<0.05, **p<0.01, ***p<0.001.

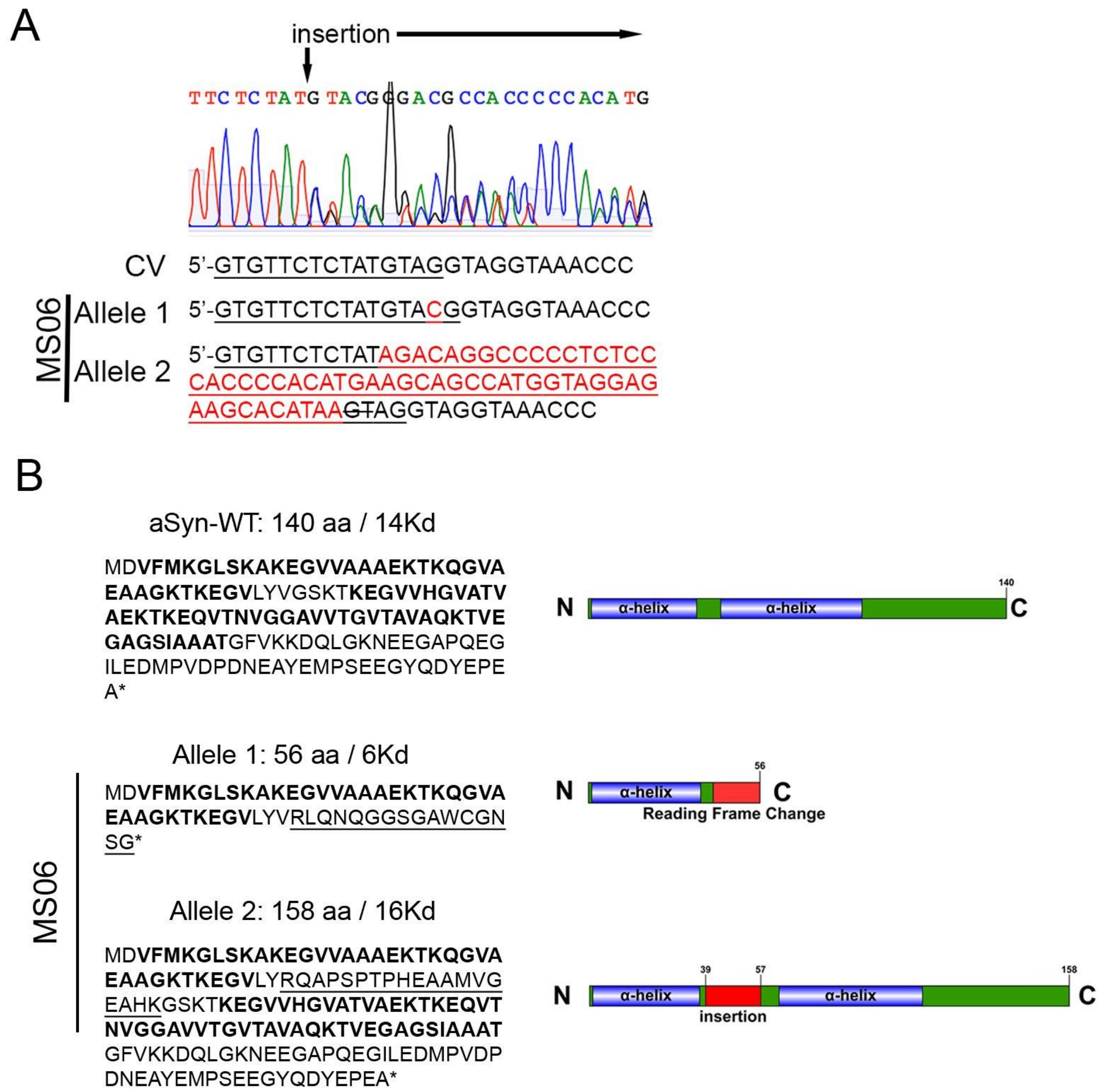

Fig.S7. αSyn disrupted sequence. Related to Figure 5. (A) MS06 genomic sequencing revealed insertions in both alleles (red colored nucleotides). Exon 2 target sequences is underlined and showing an insertion before the last nucleotide in one allele and an in/del event of 56 nucleotides insertion plus 2 nucleotides deletion (crossed). (B) Amino acid sequence of WT αSyn and MS06 CRISPr/Cas9 modified alleles. In bold are amino acids that correspond to the two N-terminal α-helix. Underlined are the change in reading frame for allele 1 and the insertion for allele 2 (in red in the scheme).

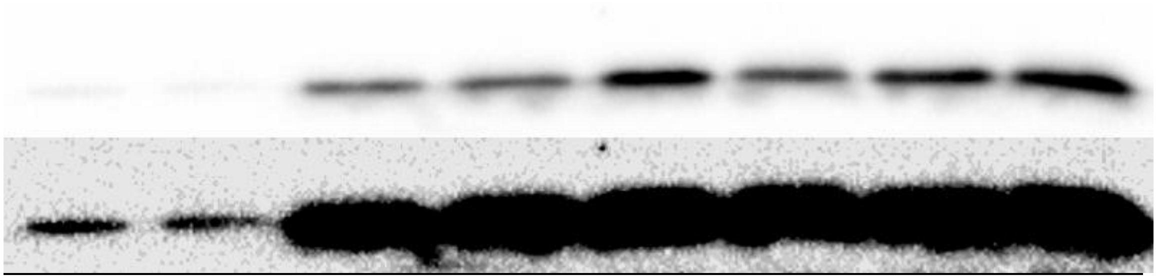

Fig.S8. Original Blots corresponding to Fig. 4A, blotted against αSyn. Upper panel: normal exposure, lower panel: overexposure.

Supplemental Movies Legends

Movie 1. Mitochondrial movement of cherry transfected neurons. Related to Figure 2. Representative time-lapse movie of mito-GFP tagged mitochondria on cherry transfected neurons, at one frame per 3 seconds for 150 seconds. Scale bar: 10μm.

Movie 2. Mitochondrial movement of WTαSyn transfected neurons. Related to Figure 2. Representative time-lapse movie of mito-GFP tagged mitochondria on WTαSyn transfected neurons, at one frame per 3 seconds for 150 seconds. Scale bar: 10μm.

Movie 3. Mitochondrial movement of A30PαSyn transfected neurons. Related to Figure 2. Representative time-lapse movie of mito-GFP tagged mitochondria on A30PαSyn transfected neurons, at one frame per 3 seconds for 150 seconds. Scale bar: 10μm.

Movie 4. Mitochondrial movement of A53TαSyn transfected neurons. Related to Figure 2. Representative time-lapse movie of mito-GFP tagged mitochondria on A30PαSyn transfected neurons, at one frame per 3 seconds for 150 seconds. Scale bar: 10μm.
